# Understanding words in context: A naturalistic EEG study of children’s lexical processing

**DOI:** 10.1016/j.jml.2024.104512

**Published:** 2024-03-08

**Authors:** Tatyana Levari, Jesse Snedeker

**Affiliations:** Department of Psychology, Harvard University, United States

**Keywords:** N400 Response, Lexical access, Natural listening, Children’s language comprehension, Discourse processing, ERPs

## Abstract

When listening to speech, adults rely on context to anticipate upcoming words. Evidence for this comes from studies demonstrating that the N400, an event-related potential (ERP) that indexes ease of lexical-semantic processing, is influenced by the predictability of a word in context. We know far less about the role of context in children’s speech comprehension. The present study explored lexical processing in adults and 5–10-year-old children as they listened to a story. ERPs time-locked to the onset of every word were recorded. Each content word was coded for frequency, semantic association, and predictability. In both children and adults, N400s reflect word predictability, even when controlling for frequency and semantic association. These findings suggest that both adults and children use top-down constraints from context to anticipate upcoming words when listening to stories.

## Introduction

A central part of language comprehension is recognizing words, retrieving their meanings, and integrating them into the discourse you are hearing. When we hear a word out of the blue, with no prior context, we often identify it bottom-up, using only the sound of the word itself. More typically, however, word comprehension occurs in the context of a sentence or a conversation that can guide us to the correct meaning before the word is fully produced. For example, we can infer that the statement “*He made a peanut butter and jelly*…” is likely to end with “*sandwich*.” Adults rapidly use top-down cues to disambiguate and predict upcoming words (e.g. Altmann & Mirković, 2009; Federmeier, 2007; Kuperberg & Jaeger, 2016; McRae et al., 2005; McRae & Matsuki, 2013), but we know very little about how this ability develops. The bottom-up process of mapping speech sounds to words has been extensively studied in infants and children. It develops early and becomes more rapid and efficient during childhood (Fernald et al., 2001; Sekerina & Brooks, 2007; Swingley et al., 1999). In contrast, there are few studies on children’s use of top-down constraints during lexical processing and the findings are mixed (Henderson et al., 2013; Khanna & Boland, 2010). Some studies have found that children make less use of top-down information than adults, both in the resolution of lexical ambiguity (Rabagliati et al., 2013) and syntactic ambiguity (Kidd & Bavin, 2005; Snedeker & Trueswell, 2004; Snedeker et al., 2009). This pattern of findings suggests that there might be a developmental shift from bottom-up to top-down processing as children acquire knowledge about the world and become more adept at coordinating these information streams (Snedeker & Huang, 2015; Yacovone et al., 2021b).

The present study uses EEG to explore the comprehension of spoken words in adults and children between the ages of five and ten, in a rich and ecologically valid context. We look at the N400 response, a negative going deflection in the event-related potential (ERP) that typically peaks around 400 ms after word onset. The N400 response in adults is sensitive to a wide range of factors that affect the processing difficulty of a word in context and has therefore been argued to reflect the ease of lexical processing (e.g. Kutas & Federmeier, 2011; Van Petten, 1993; Van Petten & Luka, 2006).

Our task (the Storytime Paradigm) is an adaptation of a method that has been used to study language comprehension in adults (Alday et al., 2017; Brennan et al., 2016; Brennan & Hale, 2019; Zhang et al., 2021) and more recently, to study syntactic processing in children (Brennan et al., 2019).^1^ Participants listen to a story while we collect continuous EEG. We then model the event related response to each content word in the story to draw inferences about the role of top-down constraints during lexical comprehension. In the remainder of this Introduction we 1) describe what we know about lexical access in adults; 2) discuss the functional characterization of the N400 in adults 3) review the prior work on lexical access in children, noting the absence of strong evidence for the use of top-down lexical prediction, 4) discuss the limited characterization of the N400 in young children and finally 5) describe our method for looking at lexical access during naturalistic listening and introduce three possible hypotheses about children’s use of top-down context.

### Lexical access in adults

To understand speech, we must construct a series of representations (phonological, syntactic, and semantic) that link sounds to conversational intentions. This process is incremental: as we hear each speech sound, we update our hypotheses about the word that is being spoken, activate the meanings of those candidate words, and begin integrating these potential meanings into the sentence (e.g. Allopenna et al., 1998; Lee et al., 2012; MacDonald et al., 1994; Yee & Sedivy, 2006). This flow of information from lower to higher levels of representation is called *bottom-up processing*. Critically, information also flows in the other direction: as adults are listening to a sentence, their knowledge of the speaker’s intentions and likely sentence meanings shapes their hypotheses about upcoming words and sounds. This flow of information is often referred to as *top-down processing* (e.g. Kutas & Federmeier, 2011; Nieuwland & Van Berkum, 2006; Van Berkum et al., 2005; Van Berkum et al., 1999).

In the present study, we focus on one step in language comprehension: retrieving the meaning of a spoken word, here referred to as lexical access. As a word unfolds, we incrementally map the sounds we are hearing onto stored lexical representations. The degree to which ease of lexical access is determined by bottom-up vs. top-down processing depends on the amount of noise in the perceptual signal, the context in which a word is found, and the degree to which that context constrains the meaning of the word.

When comprehenders encounter a word in isolation, frequency plays a critical role in lexical access: more frequent words are identified more quickly and on the basis of less perceptual information (for review see Brysbaert et al., 2018) which affects naming times (e.g. Jescheniak & Levelt, 1994; Oldfield & Wingfield, 1965), lexical decision times (e.g. Gimenes & New, 2016), reading times (Gerhand & Barry, 1998), referent identification (Erker & Guy, 2012), and length of fixations on a word during reading (Kretzschmar et al., 2015; Staub, 2015). In EEG studies of isolated words, more frequent words have smaller N400 responses suggesting that they are more easily processed (Dambacher et al., 2006; Halgren & Smith, 1987; Rugg, 1990; Van Petten, 1993). Because frequency effects on single word comprehension are strong and ubiquitous, frequency was built into the bones of many early lexical processing models. For example, in many spreading activation models, each word has a set threshold which determines the level of activation required before it will be recognized (Marslen-Wilson, 1990; McClelland & Rumelhart, 1981; Morton, 1969). These thresholds are an inverse function of frequency, such that more common words have lower thresholds and thus require less perceptual input (and less time) for recognition (Solomon & Postman, 1952).

Early research on lexical processing in adults found that speed of access is not only affected by properties of the word itself but can also be influenced by the words that precede it. The classic demonstration of this is semantic priming: we are faster and more accurate in identifying a word (e.g. *cat*) when it is preceded by a related word (e.g. *dog*) than when it is preceded by an unrelated word (e.g. *chair*) (Meyer & Schvaneveldt, 1971; Moss et al., 1995; Swinney, 1979). Latent Semantic Analysis (LSA) measures have been used to measure lexical associations across words by tracking the contexts in which a word occurs and calculating the degree to which two (or more) words share the same contexts (Landauer & Dumais, 1997). This measure predicts the ease of lexical access as indexed by priming effects in lexical decision tasks (De Wit & Kinoshita, 2014; Günther et al., 2016; Vigliocco et al., 2009, but see Hutchison et al., 2008). LSA also predicts the magnitude of the N400 (Van Petten, 2014). Perhaps the strongest evidence that semantic priming plays a role in processing words in sentences comes from studies of cross-modal priming using homophones. Both meanings of a homophone are usually active immediately after the word is spoken, even when syntactic constraints rule out the irrelevant meaning. However, if one of the words in the prior context has a strong lexical association with the correct meaning, then the incorrect meaning is sometimes suppressed (Seidenberg et al., 1982). For example, hearing “*the farmer bought the straw*” does not prime “*soda*”.

When words are presented in the context of a sentence, passage, or conversation, lexical access can be constrained by higher-level representations of the unfolding discourse (e.g. McRae et al., 2005; McRae & Matsuki, 2013). The central role of top-down processing is supported by research demonstrating that a constraining sentence or discourse facilitates the identification and integration of words that are congruent with that discourse. Predictable words are identified faster (Craig et al., 1993), are more resistant to noise (Kalikow et al., 1977), show faster first-pass reading times (Kliegl et al., 2004; Rayner et al., 2011), and are more likely to be skipped over in reading (Rayner et al., 2004; Rayner et al., 2011). Conversely, words that are inconsistent with a preceding discourse are read more slowly (Van Berkum et al., 2005). Much of the evidence for top-down lexical processing has come from work looking at the N400 ERP response (Nieuwland & Van Berkum, 2006; Van Berkum et al., 1999, see Kutas & Federmeier, 2011 for review).

### The functional characterization of the N400 in adults

Historically there have been three ways of thinking about the N400. An early theory conceptualized the N400 response as an index of bottom-up lexical access (Holcomb & Neville, 1990). This reflected data primarily from words in isolation which showed effects of bottom-up constraints such as word frequency (Rugg, 1990; Smith & Halgren, 1987) and lateral activation due to semantic priming (Holcomb & Neville, 1990). Subsequent research on the N400 response, however, has shown that when words are presented within a sentence, the N400 response reflects top-down word predictability rather than bottom-up lexical properties (Dambacher et al., 2006; Van Petten & Kutas, 1990).

These top-down effects on the N400 gave rise to a second theory: that the response reflected later integrative stages of lexical processing (Brown & Hagoort, 1993; Burnsky et al., 2023; Kretzschmar et al., 2015; Staub, 2015). This hypothesis is primarily supported by recent data suggesting that, in sentence contexts, initial fixations to written words remain sensitive to bottom-up lexical constraints but that the N400 response is only sensitive to predictability, suggesting that the N400 reflects a later stage of processing than the fixations (Burnsky et al., 2023; Kretzschmar et al., 2015). While the early bottom-up lexical access theory of the N400 response failed to account for the top-down effects of context, the purely integrative account fails to explain why the N400 response can, under certain contexts, reflect bottom-up constraints (Nour Eddine et al., 2023).

A third theory of the N400 seeks to account for both of these effects, by positing that the response reflects lexical access, but that lexical access is not a feed-forward process but instead is one that is often guided by top-down constraints from the outset (Kutas & Federmeier, 2000; Lau et al., 2009, for review see Kutas & Federmeier, 2011). On this theory, all words produce an N400 response, which reflects the process of accessing the word or concept. When the prior context allows us to predict semantic features or words, the N400 is smaller (Kuperberg et al., 2020) and the word is easier to retrieve. In the current study, we conceptualize the N400 response in accordance with this third theory. However, in the Discussion we will consider how the current results may be interpreted under different functional characterizations of the N400.

This top-down predictive theory readily captures the findings from a wide range of N400 studies (see Kutas & Federmeier, 2011, for review). N400s are strongly and linearly correlated with cloze probability, or the likelihood that a respondent can guess a correct word given the prior context (Kutas & Federmeier, 2011; Wlotko & Federmeier, 2012). The relationship between the N400 response and word predictability persists even when constraints from semantic association are controlled for, indicating that the cloze effects cannot be explained by lateral priming alone (Rabs et al., 2022).

In adults, the N400 is sensitive not only to sentence context, but also to the context created by the broader discourse in which the sentence occurs. For example, Van Berkum et al. (1999) had participants read sentences like [1], both in isolation and in contexts which made one of the final words more coherent than the other.

[1] *Jane told the brother that he was exceptionally* quick/slow.

In isolation, both completions produced similar responses. But in a richer discourse, the N400 was smaller for the continuation that was consistent with the story (e.g. “*quick*” in a story where the brother completes a task in a shorter time than had been predicted). In fact, Nieuwland and Van Berkum (2006) showed that a supportive context can even eliminate N400 effects to verb-object animacy violations. In a story where a peanut was a sentient character, capable of emotion, the sentence in [2a] elicited a smaller N400 than the sentence in [2b].

[2a] The peanut was in love.
[2b] The peanut was salted.

When words are embedded in a context, predictability effects on the N400 response are highly robust. In contrast, frequency effects are either limited to words that appear very early in a sentence or are entirely absent (Payne et al., 2015; Van Petten & Kutas, 1990). This pattern suggests that when words are presented without relevant context (as in word lists or at the beginning of a sentence) then lexical access, as indexed by the N400, primarily reflects bottom-up activation with little help from top-down cues, resulting in frequency effects. However, when words are presented in a richer context, top-down constraints are prioritized and word meanings are preactivated, minimizing the role of bottom-up cues (Degno et al., 2019; Kretzschmar et al., 2015; Kutas & Federmeier, 2011; Payne et al., 2015; Van Petten & Kutas, 1990).

### Lexical access in children

Like adults, children access words incrementally: as the first sounds of a word are spoken, they begin activating possible lexical items and integrating those candidate words into their interpretation of the sentence (Fernald et al., 2001; Huang & Snedeker, 2011). For example, 5-year-olds, like adults, will shift their gaze to a picture of a candle upon hearing the first phonemes in “candy” but will favor the correct target after the second syllable. This ability to use bottom-up information in an incremental fashion emerges and improves in the second year of life (Fernald et al., 2001; Swingley, 2009; Swingley & Aslin, 2000). There is one way in which incremental processing is different in children than in adults: children consider competing lexical items for a longer time, suggesting that they struggle to inhibit lexical representations once they have been activated (Huang & Snedeker, 2011; McMurray et al., 2010; Sekerina & Brooks, 2007).

When words are presented in isolation, lexical access in children is influenced by many of the same variables as in adults, suggesting similar bottom-up processes (Cirrin, 1984; Plaut & Booth, 2000; Schröter & Schroeder, 2017; Smith et al., 2006; Walley, 1988). For example, six- to nine-year-old children, like adults, are slower in auditory lexical decision tasks if the words are lower in frequency or are acquired later (Cirrin, 1984). Similarly, the eye-tracking patterns of both adults (Kretzschmar et al., 2015; Staub, 2015) and children (Joseph et al., 2013; Tiffin-Richards & Schroeder, 2020) show robust effects of frequency, with more frequent words showing shorter first fixations and being skipped over more often. There is also ample evidence for semantic priming in children, providing prima facie support for models with lateral activation (see e.g. Friedrich & Friederici, 2006; Radeau, 1983; Rämä et al., 2013). For example, six- and seven-year-olds are faster to identify a word after hearing a different word from the same category (e.g. *cat* after *dog*) than they are to identify the same word after hearing an unrelated word (e.g. *cat* after *chair*) (Radeau, 1983). Semantic priming has been found in children as young as 18 months of age, both in looking time paradigms (Arias-Trejo & Plunkett, 2009) and in EEG studies (Friedrich & Friederici, 2006; Rämä et al., 2013).

In contrast, there are few studies that directly explore whether children use top-down constraints during lexical access. While a number of looking time studies (see e.g. Borovsky et al., 2012; Mani & Huettig, 2012; Nation et al., 2003) demonstrate that children can use context to shift their gaze to an upcoming referent (e.g. looking toward a cake after hearing “*eat*”), these studies do not distinguish between lexical prediction (pre-activating the word “*cake*”) and referential prediction (looking around for something edible). We know of only one study in children that directly and unambiguously assesses top-down lexical processing. Khanna and Boland (2010) had participants listen to sentences that ended with a homophone (e.g. *tag*) and then read aloud a target word (e. g. *grab*) that was related to one meaning of the homophone but not the other. Adults and children over 12 were sensitive to context: hearing *tag* facilitated reading *grab* when the preceding sentence picked out the semantically related interpretation (e.g. *At recess the children played tag*) but not when the context picked out the other interpretation (e.g. *Jerry was bothered by the shirt’s tag*). Critically, however, younger children (7–9 years) showed facilitation in both contexts, indicating that they had accessed both meanings of the word, failing to use the sentence context to constrain initial lexical processing. In contrast, when the context was reduced to a single word, even the 7–9 year olds activated only the correct meaning of the homophone (i.e., faster reading times for *grab* after hearing “*laser tag*” but not after hearing “*shirt tag*”).

This pattern of results suggests that, under ordinary circumstances, children younger than ten might have difficulty using the broader discourse context to guide lexical access. This could occur for two very different reasons. First, young children could have a language processing architecture that is broadly similar to adults; top-down connections between levels that would, theoretically, allow for contextually guided lexical access, but they could lack the linguistic skill to construct and use top-down representations quickly enough to guide lexical access. When the context is simplified (word pairs presented without additional context), constructing the top-down representation is easier and context effects emerge. Second, young children could have a processing system that is architecturally distinct from that of adults and older children. Specifically, the bottom-up connections and lateral connections might mature early (allowing for bottom-up processing and priming respectively) while the top-down pathways might take longer to mature. Such a developmental trajectory is not inconceivable. There is ample evidence that there are changes in the brain (synaptic pruning and myelination) throughout this developmental period and that these changes occur earlier in sensory areas and bottom up pathways (see e.g. Gogtay et al., 2004; Huttenlocher & Dabholkar, 1997).

There are, however, good reasons to be cautious when drawing strong conclusions about top-down processing from this single data point. The task that Khanna and Boland (2010) used, required simultaneous listening and reading. Seven- to nine-year-old children are just beginning to read and are likely to have more difficulty coordinating these tasks than older children and adults. Perhaps, these demands interfered with the children’s ability, or motivation, to listen attentively to the sentence context. Studies using slower-paced, simpler paradigms have found that young children can make use of sentential context to determine the meaning of novel words (Goodman et al., 1998), to interpret noisy input (Cole & Perfetti, 1980; Newman, 2004), and to choose the relevant sense of an ambiguous word (Rabagliati et al., 2013).

In the most relevant of these studies, Rabagliati et al. (2013) asked four-year-olds to select the picture that matched the final word in a short vignette. Critical items contained words that primed the wrong meaning of the homophone given the sentence context (e.g. a story with a princess and a dragon that takes place at *night*, not *knight*). They found that children have some ability to override these lexical associations -they select the right meaning 39% of the time - but they make a surprisingly large number of errors (compared to adults) suggesting that sentential context is often overridden by mere association.

Further evidence for developmental differences in the ability to use top-down cues comes from studies that look at the effect of context on reading time in the absence of ambiguity. Reading times for words in isolated sentences vary depending on whether the words in context are plausible, implausible, or anomalous. Adults generally slow down at both anomalous and implausible words (Joseph et al., 2008; Rayner et al., 2004). This pattern, particularly the difference between implausible and plausible words, is consistent with the proposal that adults use top-down cues during lexical processing. Children between the ages of 7 and 12 also show slowdowns early in processing for anomalous words, but critically, they do not show early differences between plausible and implausible words (Joseph et al., 2008). A similar pattern appears when children read short, coherent discourses. Tiffin-Richards and Schroeder (2020) tracked eye-saccades as adults and children (mean age 8.5) read short stories. They found that while adults show early and late facilitation of gaze based on contextual constraint, children show only late facilitation effects, suggesting that adult readers use context more efficiently to pre-activate lexical candidates.

In short, the evidence to date is consistent with the hypothesis that children under 12 readily make use of bottom-up cues and semantic association to constrain upcoming lexical items but are far less adept at using top-down cues from context. This same pattern appears in other linguistic processes (see Snedeker, 2013 for review). For example, while four- to six-year-olds will readily use bottom-up information such as intonation or lexical information to infer and disambiguate syntactic structure, they often fail to use higher level cues such as the plausibility of an event or the referential context in which the sentence is used (Kidd & Bavin, 2005; Kidd et al., 2011; Snedeker & Trueswell, 2004; Snedeker et al., 2009; Trueswell et al., 1999; Yacovone et al., 2021b).

### Limited characterization of the N400 in children

Our understanding of lexical access in adults has been shaped and informed by research looking at the N400 ERP response. Although children show an N400-like response that seems to index lexical retrieval, we know far less about the variables that influence this effect, and thus, far less about lexical access in children. In infancy, N400 responses are characterized by a widely distributed negativity that is often delayed and prolonged relative to adults (Junge et al., 2021). By the age of 5, children’s N400 responses are much more adult-like, but remain somewhat larger and a bit more distributed, delayed and prolonged (Atchley et al., 2006; Hahne et al., 2004; Holcomb et al., 1992). While there is some debate regarding the topography of the N400 response across development, most studies of school-aged show semantic congruency effects which are maximal over centro-parietal electrodes sites, similar to that of adults. (Holcomb et al., 1992; Juottonen et al., 1996, but see Atchley et al., 2006 showing more anteriorly distributed N400s in children). Between ages 5 and 18 the observed N400 response gradually becomes more adultlike in magnitude, timing and distribution (Holcomb et al., 1992).

The vast majority of N400 studies in children have focused on comparing responses to overt semantic disruptions: either semantically anomalous words in sentences, or a word that mismatches a prior word or picture (e.g. hearing “*dog*” after seeing or hearing “*cup*”). In the picture mismatch paradigm, N400-like responses appear as early as 12 months (Friedrich & Friederici, 2010; Lindau et al., 2017), becoming faster and more reliable over the second year of life (Friedrich & Friederici, 2004, 2005; Mills et al., 2005). In the violation paradigm, N400-like responses have been observed in 19-month-olds (Friedrich & Friederici, 2005) as well as in toddlers and preschoolers (Silva-Pereyra et al., 2005).

These responses to violations and mismatches could reflect the use of top-down constraints in lexical prediction, however, they could also reflect passive lateral priming by related words, or even a reactive response to inconsistency. For example, a large N400 response to “*He spread butter on his* *dog*” relative to “*He spread butter on his* *toast*” could reflect prediction of the word *toast* given the context of the sentence, priming of the word *toast* from the word *butter*, or a response to the wild implausibility of buttering a dog.

There are two other lines of research on children’s N400 responses, but they do not fully resolve these questions. First, by 14 months of age, the N400 in children is affected by semantic priming such that primed words have smaller N400s than unprimed words (Friedrich & Friederici, 2005, 2010; Sirri & Rämä, 2015; von Koss Torkildsen et al., 2007). This finding is compatible with two mechanisms: top-down prediction and lateral priming. In adults, the priming effects are modulated by the proportion of semantically related pairs in the study, favoring the top-down predictive account (de Groot, 1984). However, no parallel studies have been done in young children.

Second, Benau and colleagues explored how 10-year-old children and adults reacted to words that were congruent, mildly incongruent, or strongly incongruent in the context of the sentences in which they were presented (Benau et al., 2011). In adults, the magnitude of the response was modulated by the degrees of semantic incongruity (congruent *<* mildly incongruent *<* strongly incongruent). Ten-year-olds showed a smaller N400 response for congruent words, but there was no difference in magnitude between the response for mildly incongruent and strongly incongruent words. It is unclear how this finding bears on our theory of lexical access in children or our theory of the N400 response. On the one hand, it could be interpreted as favoring a predictive account of children’s N400 responses (only when the prediction is met does the response decrease). On this interpretation, however, the adult response would either be entirely reactive, or would consist of two processes (a predictive lowering of the N400 for expected/congruent words and a reactive response to the strongly incongruent words). Alternatively, it could be interpreted as favoring a reactive account to incongruity with children treating improbable and impossible events similarly. Such an interpretation would be consistent with research showing that children often view the unlikely as impossible (Shtulman & Carey, 2007). Critically, both interpretations rest on a null finding, in a population that typically produces noisier data. Furthermore, we have no data on how children under 10 would react to different levels of incongruity.

In sum, while children show ERP responses consistent with the N400, we do not yet know what constraints on lexical access are being captured by this response. To the best of our knowledge there is no work looking at whether the N400 in children is sensitive to word frequency or predictability per se. One aim of the current study is to determine if there is functional continuity in the N400 response in children and in adults.

### A child-friendly approach to ERP studies

In the current study we investigate the degree to which the N400 response in adults and children reflects the frequency, semantic association, and top-down predictability of an uttered word in order to investigate the sources of information that influence lexical access, as indexed by the N400 response. In a departure from prior work, we investigate lexical access in the rich, naturalistic context of a children’s story using single-trial ERP recordings.

Most EEG studies of language have used a traditional trial-based design, with many items in each condition, which are then averaged together. In these studies participants listen to, or read, a series of unrelated words or sentences, one after the next. To reduce the noise in the EEG signal, studies with adult participants often use 30–50 or more items per condition, resulting in experiments that can last for one to two hours (Luck, 2005). Adults typically comply with these demands. Children, however, are either less capable or less willing to sit still and attend to these long streams of disconnected stimuli. Researchers have adapted to the challenge of trial-based EEG testing in children by including distractor tasks (e.g. watching a silent video), reducing the number of items in each condition, tolerating more noise, and using designs with fewer conditions (typically 2) (Atchley et al., 2006; Benau et al., 2011; Pijnacker et al., 2017). As a result, many EEG studies with children are underpowered with minimal designs that leave many questions unanswered.

These designs also have an additional limitation. While trial-based EEG studies provide a time-sensitive index of lexical processing, they assess comprehension in one particular context (a list of disconnected sentences or words) which has little ecological relevance. Ultimately, we want to understand how children interpret words and sentences in real world contexts such as stories, conversations, or lessons.

The present study uses recent innovations in EEG methods to create a task that taps into children’s intrinsic motivations and produces a much denser data set, resulting in approximately 25 times as many observations in a session as a trial-based study of the same length. The first critical difference is that we are using a single-trial ERP design. In this procedure, participants read or listen to sentences as ERP responses to every word are recorded (Brennan et al., 2016; Payne et al., 2015). Each word is coded for factors of interest (e.g. frequency or predictability) as they vary across the stimuli. These factors are then evaluated as continuous predictors of N400 amplitude. This allows for a second, critical difference in the method. Since single-trial ERPs are not restricted to a trial structure, these responses can be recorded in response to naturally occurring, meaningful language such as a story or dialog.

Single-trial ERP studies with adults have replicated some of the data patterns found in traditional trial-based designs. For example, single trial designs have found that the N400 response is modulated both by low-level features such as word frequency and by higher order processes associated with lexical prediction (Payne et al., 2015). Computational models applied to single-trial ERP data have found that the N400 reflects measures of lexical predictability given the preceding context (e.g. Frank et al., 2015). Several studies have specifically argued that lexical access in adults takes into consideration top-down constraints from the hierarchical syntactic structure (Brennan et al., 2016; Fossum & Levy, 2012; but see Frank & Bod, 2011; Frank et al., 2015). While many of these trial-based designs use isolated sentences presented visually word-by-word, this method has also been applied to auditorily presented stories (Brennan et al., 2016).

In the current study, we adapt this natural story-listening paradigm for use with children. Participants listen to a story as ERPs time-locked to the onset of every word are recorded, allowing us to gather data from hundreds of trials in a task that is short and fun, making it suitable for both adults and children. Critically, this method allows us to study children’s language comprehension with greater ecological validity than other temporally sensitive methods. By using natural narratives, we can explore how children use the rich cues provided in real discourse to guide comprehension in real time, using a task which is both familiar and relevant to their lives.

The present study explores whether the cues used by five- to ten-year-old children to access upcoming words in naturalistic language input differ from those used by adults: looking first at the role of bottom-up features, specifically word frequency, then at lateral semantic activation, and finally top-down predictions based on context. While there is ample evidence that adults use this type of prediction during sentence comprehension, we know of no evidence that children of these ages do so.

We chose to focus on children of this age for three reasons. First, we suspected that five-year-old children would be able to listen to a story for at least 20 min without becoming restless. In talking with parents, we confirmed that this is the age at which many of us begin reading chapter books to our children. Third, as we noted above, previous behavioral research has found that children across this age range are less apt to use top-down context to guide lexical access in sentence contexts: 7–9-year-olds activate contextually inappropriate meanings of homophones in sentence contexts (Khanna & Boland, 2010); 7–10-year-olds do not slow down when reading unpredictable words (Joseph et al., 2008; Tiffin-Richards & Schroeder, 2020); and 10 year olds show reduced context sensitivity in EEG measures (Benau et al., 2011).

We will evaluate children’s ability to use contextual information during online sentence comprehension by seeing whether increasing use of context predicts the size of the N400 above and beyond models based on bottom-up activation alone. One possibility is that children’s lexical access is primarily driven by bottom-up constraints such as word frequency or semantic association, consistent with prior work showing a general difficulty using top-down constraints during online comprehension. On the other hand, it is possible that given a rich discourse and a natural task, both children and adults may be able to recruit top-down constraints to inform their comprehension. While this study was not designed to look for differences within the children, we also conducted exploratory analyses to see whether children at the younger end of the age range (~5–7) differed from children at the older end (~7–10).

## Methods

### Participants

We tested 27 children, aged 5–10 (mean age = 7.5) and 21 adult (mean age = 22.4) native English speakers. An additional 8 adults and 15 children were tested and excluded due to excessive artifacts (trial loss of more than 50 %) (N = 4 adults, 10 children) or technological errors during recording (N = 4 adults, 5 children). All participants had normal or corrected-to-normal vision. Adult participants were recruited from the Harvard University student body and received course credit for participation. Child participants were recruited from our database of families in the greater Boston area. Informed consent was obtained from all participants.

### Materials

Participants listened to an excerpt from Chapter 7 of *Matilda*, by Dahl (2003). The excerpt contains 1594-word tokens of 580 types, out of which 766 tokens of 444 types are content words. The story was recorded at a comfortable reading pace by a female speaker. The onsets of all words in the story were hand coded. In addition, the onsets and offsets were timed using the Gentle online text to speech aligner (https://lowerquality.com/gentle/) (Ochshorn & Hawkins, 2017). The human and computer coded onset times were highly similar (mean difference = 0.013 sec, 97 % of words *<* 0.1 sec difference).

### Procedure

Following the informed consent procedure, participants were fitted with an EEG cap, seated in front of a computer screen in a quiet testing room, and asked to listen to a story. They were told to try to remain still and keep their eyes directed at the screen. Pictures from the book were shown throughout the story. The images showed characters from the story and changed approximately every 100 words. The images were presented in a randomized order and were chosen because they had no strong connection to the events at any given moment in the story. Subjects were told that they would be asked questions about the story at the end of the session and were asked 5 recall questions.

### EEG recording and processing

All subjects’ EEG data were recorded at 500hz using Brainvision’s Actichamp System with 32 active electrodes placed at International 10–20 System locations and on the left and right mastoids. Impedances were kept below 25kOhm for all relevant electrodes. A pair of passive electrodes connected to the BIP2AUX adapter were attached to the left eye to monitor for vertical eye movements. Offline, the EEG signal was resampled to 200hz and re-referenced to the average of the left and right mastoids. EEG signals were filtered using an IIR filter with a bandwidth of 0.1–30 Hz. Data were epoched from − 200 ms to 1000 ms and baseline corrected using the pre-stimulus time window (−200–0 ms). Eye artifacts were removed through independent component analysis. Trials were discarded if they contained voltage greater/less than 100 μV and for excessive noise based on visual inspection. Offline processing was conducted using EEGLAB (Delorme & Makeig, 2004) and ERPLAB (Lopez-Calderon & Luck, 2014) software programs.

Our analyses examined the factors that predict the magnitude of the N400 effect. We operationalize N400 size as the mean amplitude between 350 and 550 ms averaged across the midline electrode sites (Fz, Cz, Pz and Oz) based on maximal expected N400 response locations based on prior literature (Duncan et al., 2009; Hagoort & Brown, 2000). Prior to running our critical analyses, we compared the voltage in the region of interest across electrode sites and their interaction with our key factors (included in Results Section). The interactions in this analysis motivated a further breakdown of the data into the canonical centro-parietal N400 measurement averaged across Pz, Cz, and Oz electrodes and an anterior effect measured at Fz.

### Measures

For each word in the discourse, we collected measures for our three critical variables (frequency, semantic association, and discourse cloze) and two control measures (concreteness and acoustic length).

#### Frequency:

Frequency was calculated as log-transformed (log10) Global Frequency (per million words) from the SUBTLEX_US_ corpus (Brysbaert & New, 2009). This is a 51-million-word corpus based on American television and film subtitles. Thus, it captures language from aurally presented narratives and conversations that are intended for a wide age range.

#### Semantic-Association:

In order to estimate semantic fit between words we used Latent Semantic Analysis (LSA) (Landauer et al., 1998; Wolfe & Goldman, 2003). LSA values were calculated based on the average co-occurrence values between the target word and any content words immediately preceding it (within 3 words). Preliminary analyses were also conducted using LSA values calculated from any immediately preceding words (content or function), as well as all the content words within the relevant sentence, leading to similar result patterns.

#### Discourse Cloze:

Discourse Cloze probability estimates for each word in the story were gathered from adult participants (N = 423) on Amazon Mechanical Turk with each participant providing responses for one of twelve parts of the excerpt, resulting in ~ 30 ratings per word. Following informed consent, participants were asked to read the excerpt used in the current study. All subjects read the entire excerpt. For one section of the story (out of 12), consisting of ~ 10 consecutive sentences, participants were asked to guess words, one by one. The correct word was revealed following each guess and the subject was then prompted to guess the next word. After this critical guessing section, participants continued reading the remainder of the excerpt. Thus, participants were asked to guess each word in a sentence based on the entire available discourse up to that point. Discourse Cloze was calculated as the percentage of correct guesses of the target word. Misspelled words were considered correct if they were phonetically consistent with the target word. Only responses with correct inflection were counted as correct.

#### Concreteness:

Concreteness estimates were gathered from adult participants (N = 235) on Amazon Mechanical Turk. Following informed consent, participants were asked to rate 50 words. Each word was presented with its part of speech. Concreteness was rated on a 7-point Likert scale, ranging from very abstract to very concrete. Subjects were given descriptions of what it means for a word to be concrete (“words refer to things or actions in reality, which you can experience directly through one of the five senses”) or abstract (“words refer to meanings that cannot be experienced directly but which we know because the meanings can be defined by other words”). Each word received an average of 20 ratings, the mean of which was used as the concreteness value.

#### Acoustic Length:

Acoustic Length was calculated as the difference between the onset and offset times of all words in the story, as detected by the Gentle online text to speech aligner (https://lowerquality.com/gentle/) (Ochshorn & Hawkins, 2017).

### Results

To evaluate the role of bottom-up and top-down cues on lexical activation we followed a theory-driven hierarchical modeling approach. First, we constructed a base model (Table 1) to control for other properties of the lexical items, or the context, that could influence the size of the N400 response beyond the factors of interest. Next, we built a series of increasingly complex models that captured different hypotheses about the nature of the lexical processes captured by the N400 effect (Table 2). The first of these models evaluated effects of frequency, a primary factor in the bottom-up activation of lexical candidates. The next built upon the first, adding our LSA measure to capture potential lateral activation through semantic association. The final model added Discourse Cloze probability to model the use of top-down constraints from the preceding discourse to make predictions about the upcoming word. In each case, our primary question was whether a given model was justified over the simpler, less sophisticated, model that preceded it. For example, to see if there was evidence for the use of frequency, we compared the base (control) model to a model that included all the factors in the base model as well as frequency. This is a conservative approach; it ensures that we can only find an effect of higher-level variables (like Discourse Cloze) if they predict the N400 response above and beyond what can be predicted by lower-level variables (like frequency and LSA).

In our initial analyses, we included both the data from the children and the adults to allow for a direct comparison. For each of the three key factors, we first introduced the factor of interest (e.g. Frequency) and then explored whether it interacted with age. We followed up this combined analysis with a separate analysis for each age group using backwards regression to find the best fitting model.

To foreshadow our central findings, we find that both adults and children show robust effects of Discourse Cloze on the size of the N400, consistent with the use of top-down prediction during lexical access.

Finally, we completed exploratory analyses to look at the possibility of developmental change across the age-range of children included in the study. We repeated the analyses comparing our younger (5–7.2) and older (7.2–10) child participants.

Predictors of N400 amplitude were evaluated using linear mixed-effects models via restricted maximum likelihood estimation. Models included random intercepts for both Subjects and Items. More maximal models were not included in the primary document due to issues with convergence, but key analyses with maximal models are included in Supplemental Materials (#6). All predictors were scaled prior to analysis. No model raised concerns regarding multicollinearity (VIF *<* 2.5). All analyses were conducted with the lme4 package (Bates, Mächler, Bolker, & Walker, 2014) in the R statistical computing environment. Bayes Factor analyses were conducted on critical null results and are included in Supplementary Materials (#7).

### Location model

To ensure that we captured the N400 response, we first ran a location model on our data comparing the effects of our key factors, Frequency, LSA, and Discourse Cloze and their interactions with channel location on N400 amplitude. We find that there is a significant Cloze Discourse by Channel Location interaction (*F* = 7.67, *p <* 0.001), such that the effect at Fz differs from all the other channels. Thus, to ensure that we accurately represent the N400 response, we conducted two separate analyses. First, we analyzed the centro-posterior N400 response collapsed across Pz, Cz and Oz electrodes. Second, we ran exploratory analyses looking at the effects at anterior electrode, Fz.

### Constructing the base model

We constructed a base model to control for extraneous variables that might affect the N400 (see Table 1). Our first model included both properties of the target word (such as its concreteness, its acoustic length, the location of the word in the sentence, and the location of the sentence in the story) and key properties of the word immediately before and the word immediately after the target (such as Frequency, Semantic Association (LSA), and Discourse Cloze probability). We included this second set of predictors to control for spillover effects of the prior word, effects of the prior word on the baseline, and effects caused by early processing of the subsequent word. As can be seen in Table 1, no control factors significantly predicted N400 size. Next, we checked whether any factors in this model differed by age group but found no significant interactions. Thus, only main effects of the control variables were included in further models.

### Analysis of key factors

We first conducted a forward regression adding the factors in the order described in Table 2, from least to most interactive (see Supplemental Material 1 for full model outputs).

### Bottom-Up frequency

**Fig. 1.**
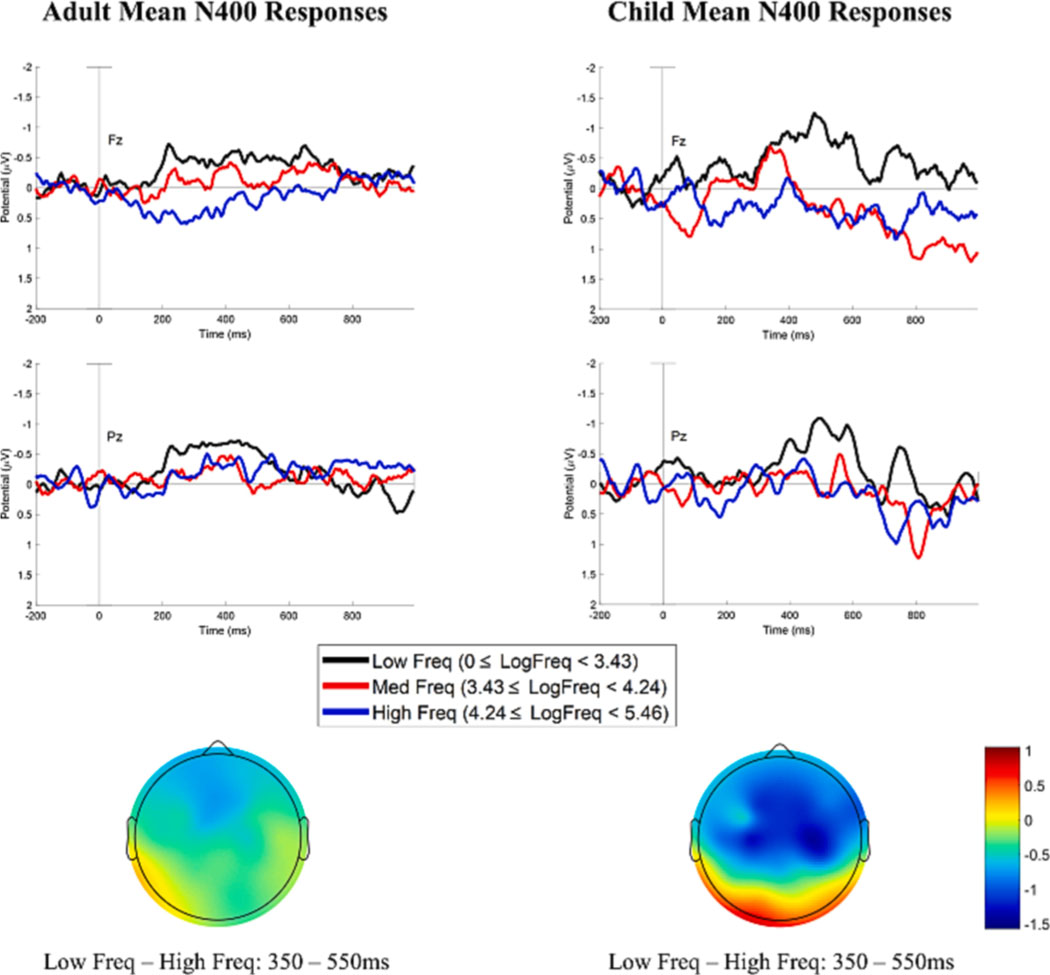
Grand average waveforms to low (*<*3.4 log freq. per million), middle (3.4 – 4.2), and high frequency (*>*4.2) words at frontal electrode Fz and centro-parietal electode Pz in adults (left) and children (right) and voltage maps of the mean difference between low and high frequency words in the time window of the N400 response (350 – 550 ms). No effect of LSA at centro-parietal sites but showing an anterior sustained negativity.

We first tested for effects of frequency by comparing the base model to a model with log frequency as a predictor (Model 1). Frequency marginally predicted N400 size (*β*_=_ 0.25, *SE* = 0.13, *p* = 0.06) and including frequency in the model marginally improved model fit over the base model (*χ*2(1) = 3.72, *p* = 0.05).^2^ As expected, more frequent words had smaller, more positive, N400 responses. When age is included as an interaction term (Model 2), there was no reliable Age by Frequency interaction. Neither adults nor children show robust effects of frequency (*p >* 0.1) with no significant improvement in model fit over the Base Model. The absence of a canonical cento-parietal effect of Frequency on the N400 response can be seen in Fig. 1.^3^

### Lateral semantic association

**Fig. 2.**
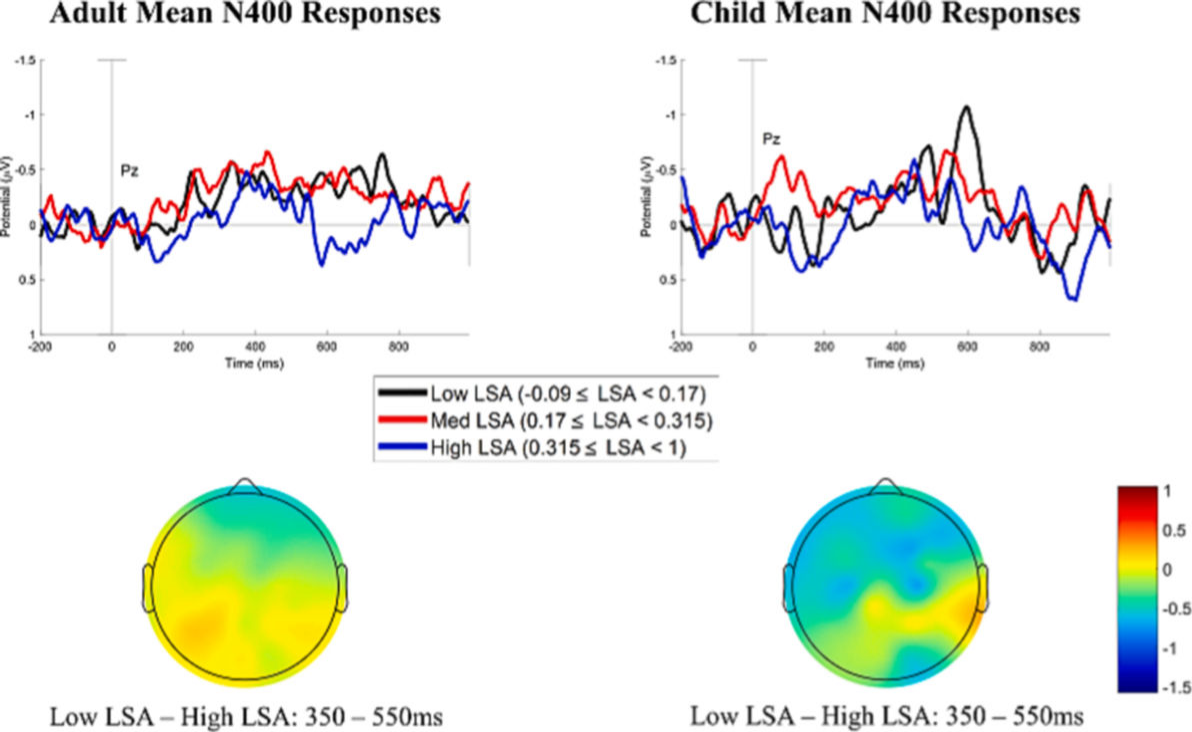
Grand average waveforms to low (*<*0.17 LSA), middle (0.17 – 3.15), and high (*>*3.15) semantic association to preceding content words at centro-parietal electode Pz in adults (left) and children (right) and voltage maps of the mean difference between words with low and high semantic association to preceding words in the time window of the N400 response (350 – 550 ms). No effect of LSA at centro-parietal sites.

To test whether semantic association contributed to lexical prediction, we looked at whether LSA predicted N400 size above and beyond the effects of Frequency (comparing Model 3 to Model 2). We found that LSA did not predict N400 size: the model with LSA did not provide a better fit to the data and the effect for LSA in this model was not reliable (See Fig. 2). Furthermore, there was no interaction between LSA and age (Model 4), and neither adults nor children showed reliable effects of LSA. Thus, in a rich discourse context, this measure of lexical co-occurrence does not appear to influence the N400.

### Discourse-Level prediction

**Fig. 3.**
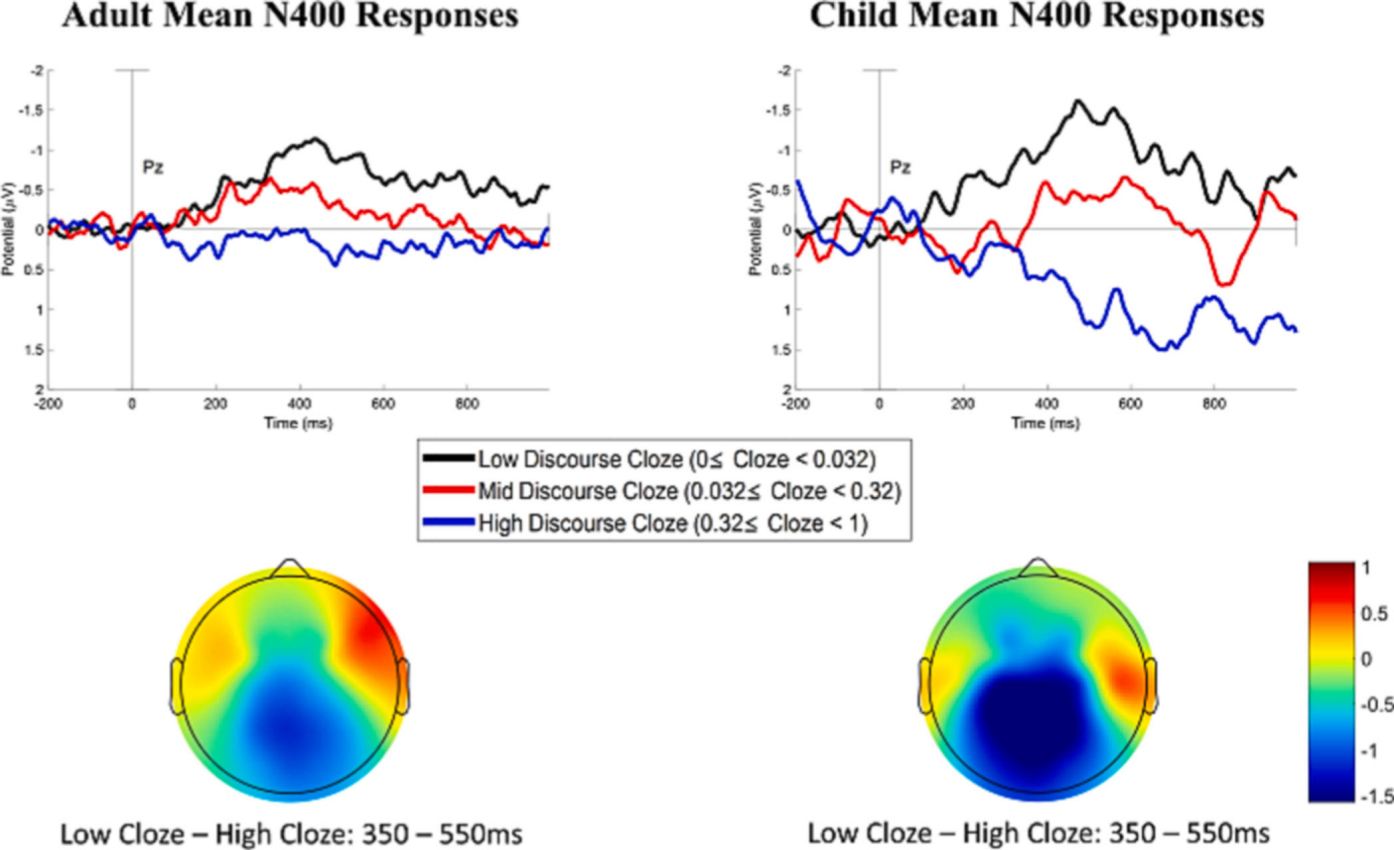
Grand average waveforms to low (*<*0.03 cloze probability), middle (0.03 – 0.3), and high predictability words (*>*0.3), as measured by Discourse Cloze probability, at centro-parietal electode Pz in adults (left) and children (right) and voltage maps of the mean difference between low and high predictability words in the time window of the N400 response (350 – 550 ms). Both adults and children show robust effects of Discourse Cloze.

In evaluating top-down constraints on lexical access, we compared the model with LSA and Frequency and their interactions with age (Model 4) to a model with Discourse Cloze included as an additional predictor (Model 5). Discourse cloze probability significantly predicted N400 size (*β*_=_ 0.48, *SE* = 0.11, *p <* 0.001) and increased model fit (χ2 (1) = 18.04, *p <* 0.001) over the simpler model. As can be seen in Fig. 3, more predictable words (words with higher discourse cloze values) show smaller N400 responses. When age is included as an interaction term (Model 6), we see no differences across the two age groups. Looking at each group separately, Discourse Cloze predicts N400 size in adults (*β* = 0.30, *SE* = 0.10, *p <* 0.01) and in children (*β* = 0.63, *SE* = 0.18, *p <* 0.001).

Since LSA did not significantly predict N400 size, we ran an additional model comparison to see whether including Discourse Cloze as a predictor improved model fit beyond the best prior model, which contained Frequency (Model 1). Including Discourse Cloze as a predictor also significantly improved model fit over the simpler Frequency Model (*χ*2(1) = 18.97, *p <* 0.001).

In all of the models described, Frequency no longer predicts N400 size when Discourse Cloze is included in the model. This pattern suggests that, in this rich discourse context, frequency has no effect on the lexical processes captured by the N400, once we account for the effect of frequency on prediction itself.

### Backwards regression

In addition to performing a hypothesis-driven forward regression, we also performed a backwards regression separately on the adult and child data. The goal of this analysis was to find the best model fit for each age group and to reduce the risk of overfitting the data sets by including too many factors in the model. The starting point for this analysis, in each age group, was a model that included all the control variables from the Base 1 model, along with the three factors of interest: Frequency, LSA and Discourse Cloze. We removed the non-significant factors, one by one, starting with the one with the highest p-value. We stopped when we reached a point at which removing further variables led to a decrease (*p <* 0.1) in model fit (an increase in AIC).

The final model for adults (Table 3) included significant effects of Discourse Cloze (*β* = 0.33, *SE* = 0.09, *p <* 0.001). The direction of the Discourse Cloze effect was consistent with the hypothesis that top-down constraints facilitate lexical processing; the N400 was larger for words that are less predictable. The final model also included a reliable effect of the Frequency of the previous word (*β* = −0.26, *SE* = 0.09, *p <* 0.05). Baseline effects were included as controls and were not specifically predicted by the hypotheses that guided this research.

The best model for children (Table 4) contained significant effects only of Discourse Cloze (*β* = 0.81, *SE* = 0.16, *p <* 0.001). The absence of any effects of the prior or subsequent word could indicate that the N400 in this time window in children is not influenced by the processing of these words or may reflect decreased sensitivity to baseline and overlap effects due to greater noise in the children’s data.

### Exploratory analyses of higher-order interactions between key variables and age

Some prior studies of words in isolated sentences have found an interaction between frequency and discourse cloze such that low frequency words show larger effects of discourse cloze (Dambacher et al., 2006). However, these results have been inconsistent (Lee et al., 2012; Sereno et al., 2020). We had not planned on looking for this interaction, or its modulation with age, for two reasons. First, we wanted to avoid overfitting our models by looking for interactions between lexical predictors, since the number of potential interactions is quite large (eight, if we ignore baseline variables). Second, unlike trial-based designs our study has few words, if any, that are not constrained to some degree by context. Once the story begins, even the first word of a sentence might be predictable. If frequency effects are the result of predictions made in a completely neutral context, our study might lack enough of these unpredictable items for such an interaction to emerge. Nevertheless, we decided to do a posthoc test to explore this possibility. Specifically, we took our final model (Model 6) and tested first, whether there was a significant interaction between Discourse Cloze and Frequency, and second, whether that interaction differed by age group. A 2-way interaction between Discourse Cloze and Frequency was not significant, however, there was a 3-way interaction between Discourse Cloze x Frequency x AgeGroup (β _=_ 0.24, SE = 0.12, p *<* 0.05). Looking at each age group independently, we see that children show no Frequency by Discourse Cloze interaction (β = −0.26, SE = 0.21, p = 0.21) while adults show a significant interaction of Frequency and Discourse Cloze (β = 0.27, SE = 0.12, p *<* 0.05), such that higher frequency words showed greater effects of Discourse Cloze. Note, however, that this interaction is in the opposite direction of the predicted pattern. We know of no reason, theoretical or empirical, why one would expect lexical access for high frequency words to be more facilitated by context than low frequency words. This could reflect overlap between the anterior effects of frequency and the more central effects of predictability, it could be a false positive, or it could reflect nonlinearities in scaling between our predictors and the effects we are measuring.

### Exploratory analyses comparing younger and older children

**Fig. 4.**
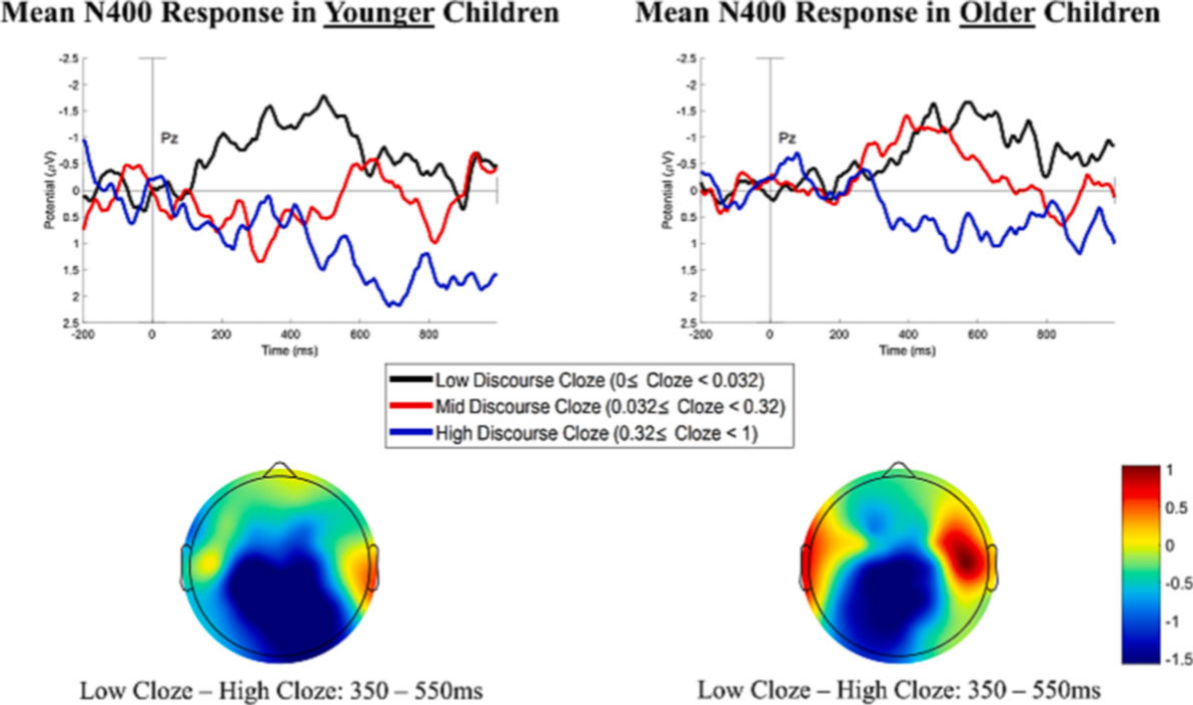
Grand average waveforms to low (*<*0.03 cloze probability), middle (0.03 – 0.3), and high predictability words (*>*0.3), as measured by Discourse Cloze probability, at centro-parietal electode Pz in Younger children (left) and Older children (right) and voltage maps of the mean difference between low and high predictability words in the time window of the N400 response (350 – 550 ms). Both groups show effects of Discourse Cloze.

The children in our study ranged from 5 to 10 years of age. This range was chosen because prior developmental studies have found limitations in top-down processing across this age range. One might wonder, however, whether there were substantial differences in our older and younger child participants. To explore this possibility, we conducted a median split on our sample of child participants, such that there were 14 younger participants (ages 5–7.2) and 13 older child participants (ages 7.2–10). Given the small sample size within each subgroup, we focused our analysis on the one variable, Discourse Cloze, which had remained in the child backwards regression analysis. Taking the final backwards model computed for child participants, we first included a main effect of Age Subgroup (Older vs. Younger) as a factor. There was no main effect of Age Subgroup on the size of the N400 response. We then looked to see if including an interaction with Discourse Cloze improved model fit. We found that there was no interaction between Discourse Cloze and Age Subgroup (*p* = 0.11). Looking separately at each Subgroup, we see that both Younger (*β* = 0.58, *SE* = 0.22, *p <* 0.01) and Older Children (*β* = 1.01, *SE* = 0.21, *p <* 0.001) show significant effects of Discourse Cloze, but the effect in Older Children is numerically larger. N400 Responses by Discourse Cloze for Younger and Older Children are presented in Fig. 4.

### Anterior effects

Our location model showed that the effect of Frequency interacted with Electrode Channel, such that the effect at Fz differed from the other three electrode sites. To better understand this effect, we re-ran our forward regression on the frontal electrode site, following the same steps described above. The base model showed no significant main effects of any control variables, however, baseline effects of frequency of the preceding word differed by age group (*β* = −0.37, *SE* = 0.14, *p <* 0.01). Thus, we included all control variables and this significant interaction in the Final Base Model and all further models.

In addition to this baseline interaction, there was also a significant effect of Frequency of the current word (*β* = 0.41, *SE* = 0.15, *p <* 0.01), resulting in a significant improvement in model fit (χ2(1) = 7.72, p *<* 0.01). The anterior negativity associated with lower Frequency words can be seen in Fig. 1. Neither LSA, Discourse Cloze, nor their interactions with Age Group further improved model fit. The effect of Frequency did not differ by age; Adults show marginal effects of Frequency (*β* = 0.27, *SE* = 0.14, *p* = 0.07) and children show significant effects of Frequency (*β* = 0.54, *SE* = 0.25, *p <* 0.05) when control and baseline variables are included in the model.^4^

## Discussion

The current study explores how adults and 5–10-year-old children access words as they listen to a story. We investigated this process by collecting data on features of words and the contexts in which they appear and then using this data to predict the size of the N400 response. We found robust effects of the predictability of the word, as measured by discourse cloze, in both adults and children, indicating that both groups use top-down constraints from the discourse to access word meaning. The posterior N400 response showed no effects of either word frequency or LSA, a measure of lexical co-occurrence. There was, however, an anterior negativity that was modulated by the frequency of both the prior word and the current word.

This pattern of effects was stable across the age groups. Specifically, the effect of discourse cloze on the N400 was statistically significant in every age group we examined–adults, all children, younger children (5–7) and older children (7–10)–and it did not interact with age in any analysis. The effect of frequency on the anterior negativity also showed no interaction with age (though it was numerically smaller in adults).

In the remainder of this section, we: 1) integrate these findings with the previous work on children’s use of top-down information during language comprehension; 2) discuss the implications of our finding that N400 responses reflect expectation (cloze) and not association (LSA); 3) explore how these findings would be interpreted under alternative theories of the N400; 4) offer potential interpretations of the limited, anterior effects of frequency, and 5) briefly describe the new questions that these findings raise and how this method, the Storytime Paradigm, could be of use in answering them.

### Children use top-down information during language comprehension

The central finding of the present experiment is that Discourse Cloze robustly predicts the magnitude of the N400 response in both adults and children. This effect persisted even after controlling for Frequency, Semantic Association, and host of control variables. Based on prior research on the N400 component in adults (Kutas & Federmeier, 2011; Lau et al., 2013; Nieuwland & Van Berkum, 2006; Van Berkum et al., 1999) and children (Atchley et al., 2006; Benau et al., 2011; Holcomb et al., 1992; Lindau et al., 2017) we interpret these effects as demonstrating that both adults and children can more readily retrieve words that are predictable in context.

This is novel and somewhat surprising, given earlier findings. As we noted in the Introduction, there are a number of studies showing that young children are less adept than adults at using top-down cues for lexical (Khanna & Boland, 2010; Rabagliati et al., 2013; Tiffin-Richards & Schroeder, 2020) and syntactic processing (Kidd & Bavin, 2005; Kidd et al., 2011; Snedeker & Trueswell, 2004; Snedeker et al., 2009; Trueswell et al., 1999). In contrast, the current findings are consistent with a smaller literature demonstrating that young school-aged children use top-down constraints to quickly focus on the contextually-appropriate meaning of an ambiguous word (Hahn et al., 2015).

To explore possible reasons for these divergent results, in the remainder of this section we will compare two studies of how young school-aged children interpret homophones (cases where two meanings are linked the same phonological form). Then, we will consider why the study that is most similar to the present one (Tiffin-Richards & Schroeder, 2020) finds no effects of context on early lexical processing. Finally, we return to the broad question of top-down constraints in language comprehension and consider how syntactic and lexical processes may differ.

#### Do children use top-down context to interpret homophones?

Two studies have looked at moment-to-moment comprehension of homophones in school-aged children, and reached different conclusions about the development of top-down processes.

The first of these studies, Khanna and Boland’s (2010) cross-modal reading experiment, found that 7–9-year-old children were unable to use sentential context to rapidly hone in on the correct meaning of a homophone, even though they were able to use single word contexts to do so. Given this pattern of results, one might have expected that children in our study would fail to use a rich naturalistic discourse to constrain lexical processing: after all, our participants were slightly younger on average and our discourse context was considerably more complex.

The second study is, to the best of our knowledge, the only experiment that finds evidence for robust use of contextual constraints in lexical access in early school-aged children. In a study using the visual-world eye tracking paradigm, Hahn et al. (2015) explored whether 6–9-year-olds were able to use sentential contexts to disambiguate homophones. While the paper focused on a comparison of children with and without autism, they found no differences between the groups, and it is the findings from the typically developing control group that are relevant here. Participants heard homophones in sentences that either only weakly constrained their meaning or strongly constrained it (*Jon saw/fed the* ***bat*** *in the cage*). In weakly constrained sentences, typically developing children showed increased looks to objects related to the incorrect meaning of the homophone (e.g. a baseball glove). However, in strongly constrained sentences these looks were no more common than in control sentences *(John fed/saw the* ***bird*** *in the cage*), even at the earliest time points.

These two studies are strikingly similar–both focus on homophones, use children of the same age and use similar context manipulations. The most obvious differences between the studies are the tasks. We propose that one possible reason for the limited use of top-down constraints in the Khanna and Boland (2010) study is the use of the cross-modal paradigm, which requires that children switch rapidly between two tasks (listening and reading) and relies on a skill (reading) that 7-year-olds are just starting to learn. Rapid task-switching could take up critical executive functioning (EF) resources which may limit the resources available for use of top-down constraints. Similar challenges with use of top-down constraints under EF load have been seen in studies of pragmatic inference (De Neys & Schaeken, 2007; Marty & Chemla, 2013; Stranahan, 2018). In addition, reading and listening are also different in a critical way. In reading, the entire form of a word is available at once, limiting the time during which top-down information might be used. In contrast, a spoken word unfolds over time, creating a longer window over which partial form information can interact with top-down constraints, potentially allowing context to play a greater role. Finally, the cross-modal paradigm provides little motivation for understanding the auditory sentence, since the participant’s primary task is to respond to the written words that appear. This may result in weaker representation of the meaning of the utterance, resulting in a greater degree of bottom-up processing. In contrast, in the visual-world paradigm (and the Storytime Paradigm) the child’s primary job is to understand what they are hearing.

The present data are consistent with the Hahn et al. (2015) findings but go beyond these results in two ways. First, the discourse constraints in our stimuli are created by a cumulative narrative while those in the Hahn study are created by local sentence structure (e.g. verb selectional restrictions). Second, we show that top-down constraints influence the processing of words in general (rather than homophones specifically).

Critically, our findings are inconsistent with the conclusions reached by Khanna and Boland (2010) who propose that the length or complexity of the context creates a burden on children such that they are able to succeed at using top-down constraints with one-word contexts but not with sentence contexts. If this were the case, then we would expect that children would fail spectacularly when confronted with a 20-minute-long narrative. We suspect that this hypothesis is wrong: the richness of the context isn’t a burden that makes top-down comprehension more difficult, rather, it is a resource that makes top-down processing more successful. In a typical psycholinguistic study, where the context is limited to a single sentence, a person must begin each trial by discarding their old discourse representation before they begin constructing a new reference world from scratch. In contrast, in our story, as in most natural contexts, the ideas in one sentence build upon those in the previous sentences, referring back to objects and people that have already been mentioned. This kind of discourse, with repeating references and conceptually linked events, should create a more coherent and memorable model of the situation, resulting in more robust use of top-down constraints.

#### Prior studies of discourse constraints during reading.

To the best of our knowledge, there is just one prior study that, like ours, uses naturalistic input to study top-down processes during children’s language comprehension. Tiffin-Richards and Schroeder (2020) measured reading times in adults and children as they read short stories. The critical words of the story were either highly predictable (and closely linked to the theme of the story) or unpredictable (and not linked to the theme). In adults, the degree of contextual constraint affected first-pass fixations but only for those trials where the first fixation was quite short. Adults also showed an effect of constraint on total gaze duration for trials with both short and long reading times. The authors interpret these effects as evidence that constraint influences both early predictive processes (with effects emerging within 100–200 ms after fixation) and late reactive processes. In children there were no reliable effects on first-fixations, but there was an effect of constraint on gaze duration which was driven solely by the trials with the longest durations. The authors interpret this pattern as showing that children only use context for late integrative processes, perhaps due to their limited reading proficiency.

Our findings support this carefully limited conclusion: when children are presented with language in a modality in which they have greater fluency and more experience (speech), they can use the discourse to predict upcoming words, leading to easy processing of predictable words and more effortful processing of less predictable words. It is unclear, however, whether the effects that we are seeing in the present study are more akin to the late effects observed in the reading study or the (missing) early effects. Based on the reading data one can estimate that context effects in the children started emerging around 500 ms after the child first fixated on the target word. In absolute time, this is similar to the period in which we measured the N400 (350–550 ms after word onset).

Absolute time, however, is unlikely to provide a reliable guide for how to align these two findings. Auditory stimuli, as we noted above, are drawn out over time and thus EEG effects for spoken language typically occur later and more variably than those to text. Pushing in the other direction, children’s limited proficiency with text might slow down decoding, leading bottom-up effects to emerge more slowly for text than for speech. Finally, the timeline for interpreting fixation duration as an index of linguistic processing is complicated by the fact that eye-movements themselves take approximately 200 ms to program and execute, resulting in a delay between the underlying cognitive process and the measure of interest (Allopenna et al., 1998; Matin et al., 1993). In fact, the early effects seen in adults in the reading study are so early (100–200 ms after first fixation) that it seems likely that many of these eye-movements were programmed before the observer moved their eyes to the critical word, and thus they presumably reflect parafoveal perception of the target prior to this fixation. Thus, the absence of early effects in the children could be attributable to differences in parafoveal processing during reading rather than to differences in prediction per se.

One way to explore these questions in greater detail would be to look for other indices of prediction in children’s comprehension of both speech and text. Most prior work on prediction in children has focused on prediction of upcoming conceptual content in an utterance (Mani & Huettig, 2012). For example, prior studies on form-based prediction in adults have found a decrease in the N400 response to non-words which are similar in form to a predicted word (Kim & Lai, 2012). For example, participants who read sentences like “For my birthday, my mother baked a chocolate” showed a smaller N400 when the sentence continued with *ceke* and a larger one when it continued with *tont*. If children fail to predict the form of a word, either when reading or listening, then they should have similarly large N400 responses to both kinds of nonwords.

#### Why do children fail to use top-down constraints during syntactic processing?

The present study demonstrates that in naturalistic, everyday contexts, young school aged children can use top-down information to guide lexical processing. This conclusion is supported by the Hahn et al. (2015) findings using the visual world paradigm. This pattern is in stark contrast to the prior findings on children’s use of top-down constraints during syntactic ambiguity resolution. When confronted with a syntactic ambiguity (*Throw the frog with the hat*), children will use bottom-up information such as prosody or verb co-occurrence patterns to arrive at an interpretation but they typically ignore top-down constraints such as the plausibility of the action or the need to resolve referential ambiguity (Kidd & Bavin, 2005; Kidd et al., 2011; Snedeker & Trueswell, 2004; Snedeker & Yuan, 2008; Trueswell et al., 1999; Yacovone et al., 2021b).

Broadly speaking there are three explanations for this apparent divergence, which are not mutually exclusive. First, the divergence could be uninteresting, resulting from differences in the tasks that are used, the measures that are taken, the strength of the constraints that are used, and the nature of the discourse in which the experimental items are embedded. While we cannot rule this possibility out, there are no obvious limitations in the tasks or materials that support this conjecture. These studies all use the visual world paradigm and thus involve no reading or secondary task. This same method has been used to demonstrate context effects in lexical ambiguity resolution (Hahn et al., 2015). In all of the syntactic studies, adults use the top-down constraints that the children fail to use, suggesting that the constraints are valid and the inference can be made on the fly.

Second, the divergence could reflect differences in the age groups tested. The lexical ambiguity studies have mostly focused on young school-agers (roughly 6–9) while the syntactic ambiguity studies have primarily focused on four- and five-year-olds (e.g. Snedeker & Trueswell, 2004). While there is no direct evidence that the use of top-down information in syntactic tasks improves in the early school years, there are reasons to think that it might. While five-year-olds tend to be rigid in sticking with their preferred interpretation, children around the age of eight are more flexible, suggesting that they are integrating more information into their ultimate analysis (Diehl et al., 2015; Weighall, 2008). Five-years-olds with higher executive function abilities or higher vocabularies show some degree of flexibility as well, suggesting that this may be a time of rapid developmental change (Arnon & Clark, 2011; Qi et al., 2020).

Finally, the divergence could reflect fundamental differences between syntactic and lexical prediction. Lexical items, by definition, are stable form to meaning mappings that must be stored in our mental dictionary. Consequently, prediction of lexical content can be conceptualized as the preactivation of an existing mental representation. The contextual cues that give rise to lexical prediction may be stable, learned associations between particular events (e.g. birthday parties) and particular words or concepts (e.g. cakes and candles). In contrast, syntactic structures are representations that are constructed, at least to some degree. Thus, in many contexts we may not be able to evaluate the alternatives and select between them until we have built them. The cognitive resources required to do this may make syntactic prediction more challenging for young children. For example, in trying to choose between the instrument and modifier interpretation of an ambiguous sentence like “*Tap the frog with the hat*” we are not merely evaluating the likelihood of instruments or modifiers appearing in a particular situation, we are also evaluating the plausibility of using a particular object as an instrument for a particular action (or evaluating whether a particular attribute would resolve the referent of a given noun phrase in a specific context). To do this we must have both built and evaluated that specific, lexicalized, syntactic structure. In other words, top-down syntactic parsing would typically build upon top-down lexical prediction.

### N400 responses reflect expectation, not association

One surprising finding of the present study is that we found no reliable effects of co-occurrence, as measured by LSA, on the processing of the current word in either adults or children as measured by the N400 response at centro-parietal electrode sites. This is in contrast with prior studies that have reported effects of LSA on the magnitude of the N400 in written word and sentence comprehension tasks (Paczynski & Kuperberg, 2012; Van Petten, 2014; Xiang & Kuperberg, 2015). Due to effects such as these, many EEG labs use LSA measures to confirm manipulations of semantic association and control for LSA values when constructing experiments to explore higher-level discourse constraints (e.g. Brothers et al., 2020; Chwilla et al., 2007).

The lack of an LSA effect in the present study is not attributable to the specific choices we made in how to operationalize LSA. We conducted additional exploratory analyses using a number of alternative methods of calculating LSA. We calculated it using the immediately preceding words regardless of whether they were function or content words, using all of the preceding content words in the sentence, and using co-occurrence values taken from a text corpus that was restricted to children’s reading level (see Supplementary Analyses 5). In every case, we found no effect of LSA on the N400. Furthermore, other EEG studies using isolated sentences, have found LSA effects on the N400 using measures that are very similar to one used in this study (Paczynski & Kuperberg, 2012; Vega-Mendoza et al., 2021; Xiang & Kuperberg, 2015).

Our findings are also incompatible with another obvious hypothesis about the relationship between LSA and cloze probability. Given the divergence in findings across studies, one might be tempted to argue that LSA values are merely an approximation of cloze probability: that they can predict the N400 magnitude when cloze values are not included as predictors, but that when cloze values are included in the model, LSA values fail to enter because discourse cloze takes up the variance that the two factors share. Our data do not support the simplest version of this hypothesis. In our forward analyses, LSA was added before discourse cloze, but it still failed to improve model fit (see Models 3 & 4).

Instead, our findings suggest that the context in which lexical processing occurs determines whether LSA is a good predictor of the N400. In isolated sentences (Paczynski & Kuperberg, 2012; Vega-Mendoza et al., 2021) or word pairs (Van Petten, 2014) there is a robust relationship between lexical association and the ease of lexical processing. In rich natural discourse, there is not. There are two ways to capture this distinction in a theory of language processing. First, one could imagine a dynamic language comprehension system in which the flow of information within and between levels is regulated based on the task at hand. In a rich discourse context, lateral semantic priming might be dampened and top-down input from the situation model might be dialed up. Alternatively, one could posit that a stable set of processes (and connection weights) are used in both cases but produce different patterns of correlation given different inputs. For example, lexical processing could always be largely driven by top-down prediction based on the discourse context. In isolated sentences (or word lists) these predictions will be based on generic knowledge and typical contexts in which these words occur, resulting in expectations that closely parallel LSA metrics. In connected discourse, however, these predictions will be informed by the particulars of the situation and thus will no longer closely correlate with the average patterns of use captured by LSA. We favor the second theory for two reasons: 1) It grounds the process of lexical access in its functional role (identifying the most likely word given the context). In real life comprehension there is no particular advantage to activating commonly associated words unless they are the ones that are actually going to be used in the near future; 2) It is consistent with findings showing that the magnitude of semantic priming in word lists depends on the composition of those lists (Bodner & Masson, 2003; De Wit & Kinoshita, 2014). Such findings strongly suggest that semantic priming reflects top-down prediction in addition to (or instead of) lateral association.

The hypothesis above (top-down lexical prediction is primary, LSA is epiphenomenal) makes the following predictions. First, future studies of the N400 in rich, natural discourse should find cloze effects, but no effects of LSA. Second, the correlation between LSA values and cloze values should be greater in existing studies of isolated sentences than in our study using a connected discourse. Third, when stimuli are constructed such that expectations based on top-down prediction differ from those based on association, top-down prediction should win out. Preliminary evidence for this last prediction comes from prior studies demonstrating that discourse expectations affect the N400 even when patterns of association are either controlled or stacked against top-down expectation (Nieuwland & Van Berkum, 2006; Van Berkum et al., 2005).

It is worth noting that while we did not find any effect of LSA at centro-parietal sites, we did find a small but robust effect of LSA at the anterior electrode site for children. This suggests that lateral activation may play a role in word retrieval or integration, but that discourse predictability is critical when context is available.

Finally, although we did not find effects of LSA, it is possible that LSA is a poor model of semantic fit, and a different model may have predicted the N400 response more robustly. For example, HAL (Lund & Burgess, 1996) and Word2Vec (Mikolov et al., 2013) additionally consider positional information that may better represent how our minds calculate semantic fit. Future work should utilize these methods to better assess the role of lateral semantic spread on the N400 response.

### Characterizing the N400: Are we measuring lexical access or lexical integration?

There is ongoing debate regarding the functional characterization of the N400 response (Mantegna et al., 2019; Nieuwland et al., 2020). Very early theories of the N400 conceptualized it as an index of feed-forward lexical activation with responses to words reflecting constraints from lexical properties and lateral activation (Holcomb & Neville, 1990). This hypothesis has been largely rejected in light of the growing evidence of robust effects of top-down constraints from context (Nour Eddine et al., 2023). The effects of predictability have given rise to two alternative theoretical interpretations. One theory conceptualizes the N400 as an index of a late-stage lexical process in which contextual information is integrated with activated lexical entries (Dambacher et al., 2006; Van Petten & Kutas, 1990). This integrative account of the N400 accounts for findings that show that bottom-up cues to lexical activation are minimal or fully absent when words are processed in context (Brown & Hagoort, 1993; Kretzschmar et al., 2015). This conceptualization of the ERP response is also supported by recent reading studies showing a dissociation of eye-gaze and ERP responses, with eye-gaze measures showing additive effects of both frequency and predictability, while ERP responses showing sensitivity only to predictability (Kretzschmar et al., 2015). An alternative account bridges the findings from both bottom-up and top-down effects on the N400 by suggesting that the N400 response reflects lexical activation as mediated by rapid predictive processing from top-down constraints when such constraints are available (Kutas & Federmeier, 2000; Lau et al., 2009). While we have focused on this final theory, we believe that our data is consistent with both contemporary accounts.

Under the integration hypothesis, the current findings suggest that children and adults build up contextual representations of the unfolding discourse and can integrate this top-down information with activated lexical representations. An integrative conceptualization of the N400 is consistent with our observation that there was no effect of frequency in either adults or children. If frequency effects arise at an early stage of lexical processing (one captured by initial fixations in reading studies, see (Kretzschmar et al., 2015), then they should not be present in a measure that reflects a later integrative process.

In this paper, we have interpreted the N400 modulation as evidence for the predictive use of top-down constraints, consistent with theories that argue that the N400 reflects lexical access as mediated by both bottom-up and top-down constraints, depending on the context in which they are found (Barber & Kutas, 2007; Kutas & Federmeier, 2011; Lau et al., 2009; Lau et al., 2008). Under this hypothesis, our data suggests that both children and adults can rapidly integrate top-down constraints during lexical access and prioritize such information when context is available. In addition, the current lack of frequency effects are consistent with prior work showing a lack of frequency effects on the N400 in a discourse context. Under predictive theories this is taken as evidence that bottom-up cues play a negligible role in lexical activation when top-down context is available.

The predictive theory, however, encounters problems in explaining the eye tracking findings. If top-down constraints are prioritized whenever context is available, then why do initial fixations show additive effects of both frequency and predictability? While the answer to this question is not yet clear there are a range of possibilities that would be consistent with the predictive theory. Initial fixation duration and gaze duration are measures based on the execution of a saccade planned in a neural system that is separable from lexical access. Saccadic planning takes time: if an initial fixation is around 250 ms, it is generally assumed that the saccade away from that target was planned around 100 ms after the eye landed on the word. For this reason (and others) shifts in fixation during reading are believed to be initiated at least in part based on information about the currently fixated word that was available when fixating on the previous word (see e.g., Reichle, Rayner, and Pollatsek, 1999; Engbert and Kliegl, 2001). The complex dance between ocular motor planning and lexical processing presents at least three cognitive loci for frequency effects: 1) frequency could affect the degree to which the wordform can be visually processed by peripheral vision (with more frequent words being more effectively processed); 2) frequency could affect early perceptual processes initiated at first fixation; 3) frequency could directly affect lexical access (even in sentence contexts). It is the third possibility that could be taken as evidence that the N400 does not reflect lexical access (because it does not reflect frequency in these contexts). This argument hinges on lexical access being a unitary process and a single moment in time. But if we conceptualize lexical activation as a complex process involving multiple streams of information (including a comparison of predictions with the input, as well as an estimate of the utility of continuing to look at the current word) then the divergence of the two measures, while still theory constraining, does not force us to interpret the N400 as a late integrative component.

Critically, while we cannot rule out the possibility that integration plays some role in the N400 modulations that we observed, we have three reasons for thinking that integration is not the primary driver of the observed effects.

First, our critical measure, discourse cloze, is a direct assessment of how accurately adults can produce the next word (given unlimited time) and thus has face validity as a measure of prediction. It does not take into account the degree to which unexpected words are semantically consistent with the context and thus does not have face validity as a measure of integration. Prior studies contrasting initial retrieval with integrative processes have concluded that cloze probability influences retrieval rather than integration, consistent with the interpretation that comprehenders are predictively activating candidate words or concepts in high cloze contexts (Lau et al., 2016; Mantegna et al., 2019; Nieuwland et al., 2020). Integration, in contrast, is typically studied with measures like semantic congruity or global plausibility.

Second, studies which directly pit prediction and integration find that predictive processes have a much larger effect in the N400 window than integrative processes (Lau et al., 2016; Mantegna et al., 2019; Nieuwland et al., 2020). There are a couple of reasons to think that the effects of plausibility or congruity would be even smaller in the present study. Prior studies focused solely on nouns that resulted in complete interpretable units (either noun phrases or transitive clauses). The present study looked at N400 modulation across words from various syntactic categories and sentence positions. As a result, most of the words do not complete phrases that can be evaluated as plausible or implausible. Furthermore, the prior studies used stimuli that were manipulated in a manner that resulted in considerable variation in the plausibility of the phrase (e.g. “yellow bag” vs “innocent bag”). In the present study, we present words that actually occurred in the original narrative, which should all result in sensible, coherent continuations of the discourse. Future work using our paradigm could better target retrieval vs. integration by modeling predictability, as well as additional measures of sentential constraint or degree of semantic fit.

Third, both the timing and the scalp distribution of our effects are consistent with the hypothesis that the N400 modulations reflect facilitated access for predictable words. Specifically, previous studies have found that predictable words are facilitated within about 200 ms in both reading and listening (Lau et al., 2016; Nieuwland et al., 2020; Nieuwland, 2019). In contrast, integration effects emerge later and linger longer. Prediction effects are generally largest at the central and parietal midline electrodes, while integration effects are often more frontal and asymmetric (Lau et al., 2016; Nieuwland et al., 2020). While some theorists propose that these two patterns are the result of two discrete processes, others view N400 effects as the reflection of a single continuous process with a gradually shifting set of inputs over time (see e.g. Lau et al., 2008; Nieuwland et al., 2020).

Critically, the importance of our findings does not primarily depend on the theoretical characterization of the N400. On any theory, the current paper demonstrates a functional equivalence between the N400 response in children and in adults, which had not previously been established. Research on children’s N400 responses has been far more limited than research with adults. Most work has focused solely on individual words (primed by other words or pictures). Studies of children’s N400 responses to words in sentence contexts have focused on semantic violations. In this study, we show that in children, as in adults, N400 responses to words in context are highly sensitive to the predictability of the word.

### Limited, anterior effects of frequency

We found two distinct patterns in our analyses. At the canonical electrode sites where the N400 is typically found, we saw a large effect of discourse cloze but no reliable effect of frequency. Specifically, for both adults and children, the effect of frequency is not significant once discourse cloze enters the model and this variable is not retained in the best-fit model. In contrast, at the anterior sites we observed a prolonged negativity for lower frequency words. In both adults and children this anterior negativity was not sensitive to differences in predictability. In children, however, it was sensitive to lexical association as measured by our LSA index. In this section we explore why we see no effects of frequency on the N400 and explore possible interpretations of the late anterior negativity.

Absence of frequency effects on the size of the N400 response is consistent with a growing consensus in the literature that, in adults, access to word meaning, as indexed by the N400, is dominated by top-down activation when words are presented in context (Degno et al., 2019; Kretzschmar et al., 2015; Van Petten & Kutas, 1990). As we noted above, frequency effects on the N400 are robustly present in studies that use isolated words (Barber et al., 2004; Condray et al., 2010; Grainger et al., 2012; Hauk & Pulvermüller, 2004; Lehtonen et al., 2012; Münte et al., 2001; Rugg, 1990) but are largely absent in studies that use sentential contexts (Burnsky et al., 2023; Staub, 2015). Specifically, frequency effects are often only detectable at the beginnings of sentences and may not be present at all (Brown et al., 1999; Dambacher et al., 2006; Degno et al., 2019; Fischer-Baum et al., 2014; Kretzschmar et al., 2015; Penolazzi et al., 2007; Van Petten & Kutas, 1990; Van Petten et al., 1991). In a connected narrative, like ours, even the beginnings of sentences are constrained by the preceding discourse and thus are somewhat predictable. As a result, many of the words in our study may be within the range of predictability in which frequency effects are absent. From a theoretical perspective this suggests that, in many real-world contexts, frequency in particular, and bottom-up processing more generally, may play little to no role in the lexical processes indexed by the N400.

While absence of frequency effects is consistent with prior literature in adults, these questions have not been comprehensively explored in children. Our discovery that children also show no frequency effect on N400 responses in connected discourse is inconsistent with theories that predict greater reliance on bottom-up features in children’s sentence comprehension (Huang & Snedeker, 2011; Snedeker & Trueswell, 2004; Yacovone, Shafto, et al., 2021). One might question the scope of this finding: our stimulus was a story that was intended for young children and thus it seems plausible that it might not have a sufficient number of low frequency words to produce the kinds of effects that have appeared in previous studies using single-word contexts Closer examination of our story rules out this possibility: the frequency range in the current story is comparable to studies that do detect frequency effects, with minimum frequency at 0.2 words per million and roughly a third of the items with frequencies of fewer than 50 words per million (Van Petten & Kutas, 1990). Furthermore, the observed anterior effects of frequency suggest that there is sufficient variance within our stimuli to detect frequency related differences in the ERPs. Thus we conclude that the absence of frequency effects on the N400 in both groups, suggests that top-down constraints play a central role in lexical access when words are presented in context from 5 years of age through adulthood.

While we do not see effects of frequency on the N400 response, we do see effects of frequency (and in children, LSA) on a late anterior negativity. While the N400 response indexes lexical access, the anterior negativity may index recruitment of additional resources or more difficult integration. Anterior negativities similar to the one recorded here have been previously detected to words that, for a variety of reasons, require additional working memory or updating (see Nieuwland & Van Berkum, 2008; Van Berkum et al., 1999 etc.). For example, prolonged anterior negativities have been recorded for referentially ambitious anaphors (Nieuwland & Van Berkum, 2008; Van Berkum et al., 1999), light verbs (Wittenberg et al., 2014), and words that are difficult to integrate in the context (Yacovone et al., 2021a). The anterior negativities associated with more difficult words in the current study are consistent with theories that suggest that such effects reflect difficulty in semantic processing or integration. As we noted above, studies that attempt to disentangle prediction and integration often find effects of integration on late prolonged negativities with a more anterior distribution (Lau et al., 2016; Nieuwland et al., 2020). A word that is preactivated based on top-down constraints may be easier to access, but its lexical or conceptual complexity (which is correlated with frequency) may nevertheless result in additional processing demands eliciting sustained anterior negativity.

### New Methods, new questions

The present findings raise a number of new questions. We next identify these questions and discuss how we might address them using the Storytime paradigm. We end by highlighting the advantages and limitations of our paradigm and how it might be adapted to address a wider range of questions.

First, the current findings raise questions regarding how the ability to use context to access words develops during early childhood. Prior work has found that young school-aged children, like those in our study, are quite adept at following narrative and expository discourse (Best et al., 2008; Kim, 2020; Lynch et al., 2008). They can understand stories, retell them, and draw a range of inferences from what they hear. These abilities appear to increase dramatically during between three and eight years of age, perhaps because of the tight connections between discourse comprehension and the ability to understand other people’s mental states (see e.g. Kim, 2020). Consistent with this, exploratory results in the current study suggest that although even our youngest children (ages 5 – 7.2) show effects of discourse cloze, these effects were less robust than in our older children. We expect that children’s ability to use discourse to make lexical predictions will develop in tandem with their ability to understand that discourse. The Storytime Paradigm, which allows a large amount of data to be collected in a short, child-friendly task, is well suited to investigating how these abilities emerge in younger, preschool-aged children, how they change during the school years, and the degree to which they are affected by the complexity of the text and child’s understanding of the genre and content.

Second, the current findings raise the question of when predictive lexical access may develop in children who vary in their linguistic experience and cognitive abilities. Using this paradigm, we can investigate whether the emergence of predictive ability may relate to individual differences in verbal ability, executive functioning, and/or the emergence of literacy. In addition, we can compare the degree to which individuals with different developmental histories may rely on predictive processing. For example, although adolescents with autism seem to show evidence of syntactic prediction (Brennan et al., 2019), the present paradigm can be used to assess the development of lexical prediction in this population. The Storytime Paradigm uses stories and passages that are of high interest to our participants, tapping into their intrinsic motivations (to be amused or informed). As a result, we do not need a secondary task to ensure that participants are listening to the story. Thus, the paradigm is well suited to testing a wide variety of populations, including those who have difficulty learning new tasks or attending to arbitrary stimuli (e.g. Brennan et al., 2019).

Third, prior work on children’s understanding of spoken words has focused almost exclusively on how words are processed in isolation or within short, highly limited contexts (Lindau et al., 2017). As we noted in the Introduction, many of these studies have found that young children either fail to use context or do not use it as effectively as adults. In contrast, the present study finds equally strong effects of context in adults and young children and no evidence that children rely more on low level information like frequency. One plausible explanation for these divergent findings is that children are better able to use discourse level constraints when they are doing a familiar task and the discourse is long, complex, and meaningful. When the discourse itself is incoherent (at least across items), when the task is unfamiliar, or when the communicative goals are unclear, this ability breaks down. This possibility raises further questions as to whether children’s linguistic abilities have been underestimated due to the lack of ecological validity of prior work. The current paradigm can be adapted to target other late-emerging language abilities, such as use of real-world knowledge, scalar implicature, and negation.

There is, however, one clear limitation to the paradigm we used. It is correlational rather than experimental and thus we cannot rule out the possibility that the effects that we observed were due to variables other than the ones that we measured. Perhaps there is some other factor, closely correlated with discourse cloze probability, that accounts for the observed effects of cloze on the N400. We believe that this is unlikely in the present case: cloze effects like the ones we found have been observed in experimental studies with adults (e.g. Nieuwland et al., 2018; Szewczyk & Federmeier, 2022). Although no parallel experimental findings exist for children, it is most parsimonious to assume that the very similar findings in children and adults reflect similar underlying processes. Nevertheless, experimental designs and naturalistic correlational designs have complementary strengths and pursuing both approaches in parallel is likely to produce a more stable science. Recent work in our lab has explored how to combine the ecological validity of the Storytime Paradigm with the control of an experimental design by using the natural story as a substrate for an experimental manipulation (Yacovone et al., 2021; Yacovone et al., 2022). While these designs necessarily have fewer trials, they retain the advantages of greater familiarity and more motivating content.

This experimental approach could be useful in understanding the nature of the predictions that children are making and how they facilitate lexical access. Our current findings cannot tell us whether high cloze words are easier to understand because children are predicting the sounds of the word, the identity of the word, or the meaning of the word. Our current findings also cannot tell us whether children are making a single explicit prediction or are probabilistically preactivating many compatible continuations. We can test these theories by manipulating or replacing highly predictable words to see whether the effects depend on the form of the word, the identity of the word, or merely only its meaning. Parallel studies with adults have found evidence for predictive processes at many levels (Kutas & Federmeier, 2000; Laszlo & Federmeier, 2009; Van Den Brink et al., 2001).

## Conclusions

When listening to a story, both adults and children use top-down constraints from the unfolding discourse to predict upcoming lexical items and these constraints dominate over lower-level information such as frequency or word-to-word association. While the current study demonstrates that children are sensitive to top-down constraints, as indexed by adult cloze probability values, it leaves open questions of how children make these predictions and what information sources they use to do so. The Storytime Paradigm provides a tool to explore these questions in adults, in typically-developing children, and in persons with developmental disorders.

## Supplementary Material

MMC1

## Figures and Tables

**Table 1:** Final Base Model for centro-parietal N400 Response.

Factor	Beta Estimate	Std. Error	T value	p-value
Intercept	− 0.32	0.12	− 2.67	0.01*
Age Group	− 0.05	0.11	− 0.47	0.65
Lexical Controls: Concreteness	0.14	0.11	1.27	0.21
Acoustic Length	− 0.11	0.12	− 0.96	0.34
Position in Sentence	0.02	0.11	0.22	0.82
Sentence Position in Story	0.14	0.11	1.24	0.22
Baseline Controls: Frequency N-1	− 0.20	0.12	− 1.61	0.11
Frequency N + 1	− 0.12	0.12	− 0.94	0.35
LSA N-1	0.14	0.13	1.14	0.25
LSA N + 1	− 0.08	0.14	− 0.60	0.55
Discourse Cloze N-1	0.02	0.11	0.19	0.84
Discourse Cloze N + 1	0.02	0.11	0.14	0.88

† = p < 0.1

* = p < 0.05

** = p < 0.01

*** = p < 0.001

**Table 2:** Results of Model Comparisons Predicting Mean Amplitude of the N400 in both Adults and Children.

Model	Added Factors	AIC	BIC	DF	Model Comparison
Base Model	See Table 1	166,645	166,764	15	
Model 1	+ Frequency	166,643	166,770	16	χ2(1) = 3.72, p = 0.05†
Model 2	+ Frequency x Age	166,645	166,779	17	χ2(1) = 0.27, p = 0.60
Model 3	+ LSA	166,646	166,788	18	χ2(1) = 0.98, p = 0.32
Model 4	+ LSA x Age	166,647	166,798	19	χ2(1) = 0.22, p = 0.64
*Model 5*	+ *Discourse Cloze*	*166,631*	*166,790*	*20*	*χ2(1)* = *18.04, p* = 0.000***
Model 6	+ Discourse Cloze x Age	166,632	166,799	21	χ2(1) = 1.44, p = 0.23

Factors that improve model fit are highlighted in italics.

† = p < 0.1

* = p < 0.05

** = p < 0.01

*** = p < 0.001.

**Table 3:** Best Fit Model by Backwards Regression for Adults.

Factor	Beta Estimate	Std. Error	T value	p-value
Intercept	− 0.34	0.11	− 3.18	0.004**
Discourse Cloze	0.33	0.09	3.65	0.000***
Frequency N-1	− 0.26	0.09	− 2.90	0.004**

† = p < 0.1

* = p < 0.05

** = p < 0.01

*** = p < 0.001.

**Table 4:** Best Fit Model by Backwards Regression for Children.

Factor	Beta Estimate	Std. Error	T value	p-value
Intercept	− 0.302	0.162	− 1.866	0.073†
Discourse Cloze	0.806	0.152	5.304	0.000***

† = p < 0.1

* = p < 0.05

** = p < 0.01

*** = p < 0.001.

## Data Availability

The data and code required to reproduce the above findings are available to download at provided link. https://osf.io/n8ewb/

## Footnotes

1 The Brennan study looks at how the predictability of syntax influences MEG signals in 8–12-year-old children with and without ASD. The relationship between these findings and the present research question is unclear and complex. We discuss this in greater detail in the General Discussion.

2 This effect was only trending when both random intercepts and slopes were included in the model.

3 In Supplemental Materials (#4) we include additional analyses using child-based Frequency norms and find the same pattern of results for children’s N400s.

4 Forwards regression in children, but not adults, showed a significant effect of LSA (χ2(1)=4.23, p *<* 0.05) above and beyond effects of frequency. However, the effect of LSA was not reliable in the model collapsed by age and there was no significant interaction between effects of LSA and Age Group.
