# Supplementary material for: Understanding words in context: A naturalistic EEG study of children’s lexical processing
Source: J Mem Lang. Author manuscript; Available in PMC 2024 Aug 1. (PMC11160963; doi:10.1016/j.jml.2024.104512)
Supplement: MMC1 [file NIHMS1977564-supplement-MMC1.pdf]

Supplementary Materials for:

Understanding words in context: A naturalistic EEG study of children's lexical processing

Data and Analyses can be found at:

Contents

1) *Supplementary Table 1* presents the full model output for Table 2 in the main paper.
2) *Supplementary Table 2* presents a full correlation table of the predictors used in the current paper and the predictors explored in additional analyses discussed in the Discussion section.
3) *Supplementary Analyses 3:* To ensure that our result patterns are not due to the inclusion of proper names specific to the story, processing of number words, or due to the inclusion of words that may be novel to children, we repeated the key analyses presented in the paper using subsets of the data.
4) *Supplementary Analyses 4*: To corroborate the null effects of Frequency in children in the current paper, we conducted additional analyses child-based measures of Word Frequency
5) *Supplementary Analyses 5:* To corroborate the null effects of LSA in the current paper, we conducted additional analyses using two alternative measures of LSA.
6) *Supplementary Analyses 6:* To corroborate findings from intercept only models, we reran analyses with slopes included for all key factors by Item and by Subject.
7) *Supplementary Analyses 7:* Analyses of Bayes Factors to evaluate amount of evidence for critical null effects.

Supplementary Table 1: Full model outputs for Models 1-6. Summary was presented in Table 2 of the main paper.

**Factor**	Estimate	Std. Error	p-value
	**Model 1**		
**(Intercept)**	**-0.31**	**0.12**	**0.01***
Log Frequency	*0.25*	*0.13*	*0.06†*
Age Group	-0.05	0.11	0.64
Concreteness	0.15	0.11	0.17
Acoustic Length	0.02	0.14	0.86
Location in Sentence	0.03	0.11	0.78
Sentence in Discourse	0.14	0.11	0.22
Log Frequency N-1	-0.18	0.12	0.15
Log Frequency N+1	-0.09	0.12	0.46
LSA N-1	0.15	0.13	0.24
LSA N+1	-0.14	0.14	0.31
Discourse Cloze N-1	0.01	0.11	0.95
Discourse Cloze N+1	0.03	0.11	0.79
	**Model 2**		
**(Intercept)**	**-0.31**	**0.12**	**0.01***
Log Frequency x Age Group	-0.05	0.1	0.6
Log Frequency	*0.25*	*0.13*	*0.06†*
Age Group	-0.05	0.11	0.63
Concreteness	0.15	0.11	0.17
Acoustic Length	0.02	0.14	0.86
Location in Sentence	0.03	0.11	0.78
Sentence in Discourse	0.14	0.11	0.22
Log Frequency N-1	-0.18	0.12	0.15

Log Frequency N+1	-0.09	0.12	0.46
LSA N-1	0.15	0.13	0.23
LSA N+1	-0.14	0.14	0.31
Discourse Cloze N-1	0.01	0.11	0.94
Discourse Cloze N+1	0.03	0.11	0.79

Model 3

**(Intercept)**	**-0.31**	**0.12**	**0.01***
LSA	0.14	0.14	0.33
Log Frequency x Age Group	-0.05	0.10	0.60
Log Frequency	0.22	0.13	0.10
Age Group	-0.05	0.11	0.63
Concreteness	0.16	0.11	0.15
Acoustic Length	0.02	0.14	0.87
Location in Sentence	0.03	0.11	0.78
Sentence in Discourse	0.14	0.11	0.20
Log Frequency N-1	-0.16	0.12	0.19
Log Frequency N+1	-0.08	0.13	0.54
LSA N-1	0.10	0.14	0.44
LSA N+1	-0.19	0.15	0.20
Discourse Cloze N-1	0.00	0.11	1.00
Discourse Cloze N+1	0.04	0.11	0.75

Model 4

**(Intercept)**	**-0.31**	**0.12**	**0.01***
LSA x Age Group	0.05	0.11	0.64
LSA	0.14	0.14	0.32
Log Frequency x Age Group	-0.06	0.10	0.53

Log Frequency	0.22	0.13	0.10
Age Group	-0.05	0.11	0.64
Concreteness	0.16	0.11	0.15
Acoustic Length	0.02	0.14	0.87
Location in Sentence	0.03	0.11	0.78
Sentence in Discourse	0.14	0.11	0.20
Log Frequency N-1	-0.16	0.12	0.19
Log Frequency N+1	-0.08	0.13	0.54
LSA N-1	0.10	0.14	0.44
LSA N+1	-0.19	0.15	0.20
Discourse Cloze N-1	0.00	0.11	1.00
Discourse Cloze N+1	0.04	0.11	0.75

Model 5

**(Intercept)**	**-0.32**	**0.12**	**0.01****
**Discourse Cloze**	0.48	0.11	0.00***
LSA x Age Group	0.05	0.11	0.62
LSA	0.01	0.14	0.94
Log Frequency x Age Group	-0.07	0.10	0.51
Log Frequency	0.13	0.13	0.33
Age Group	-0.05	0.11	0.64
Concreteness	0.02	0.11	0.86
Acoustic Length	0.00	0.14	0.98
Location in Sentence	0.00	0.11	1.00
Sentence in Discourse	0.08	0.11	0.46
Log Frequency N-1	-0.11	0.12	0.37
Log Frequency N+1	-0.08	0.12	0.54
LSA N-1	0.16	0.13	0.24

LSA N+1	-0.16	0.15	0.27
Discourse Cloze N-1	-0.04	0.11	0.74
Discourse Cloze N+1	0.03	0.11	0.77

Model 6

**(Intercept)**	-0.32	0.12	0.01**
Discourse Cloze x Age Group	-0.12	0.10	0.23
**Discourse Cloze**	0.47	0.11	0.00***
LSA x Age Group	0.07	0.11	0.52
LSA	0.01	0.14	0.94
Log Frequency x Age Group	-0.05	0.10	0.65
Log Frequency	0.13	0.13	0.32
Age Group	-0.05	0.11	0.67
Concreteness	0.02	0.11	0.86
Acoustic Length	0.00	0.14	0.98
Location in Sentence	0.00	0.11	0.99
Sentence in Discourse	0.08	0.11	0.46
Log Frequency N-1	-0.11	0.12	0.37
Log Frequency N+1	-0.08	0.12	0.54
LSA N-1	0.16	0.13	0.24
LSA N+1	-0.16	0.15	0.27
Discourse Cloze N-1	-0.04	0.11	0.74
Discourse Cloze N+1	0.03	0.11	0.78

† = p < 0.1, * = p < 0.05, ** = p < 0.01, *** = p < 0.001

Supplementary Table 2: Full correlation table of the predictors included in the current study.

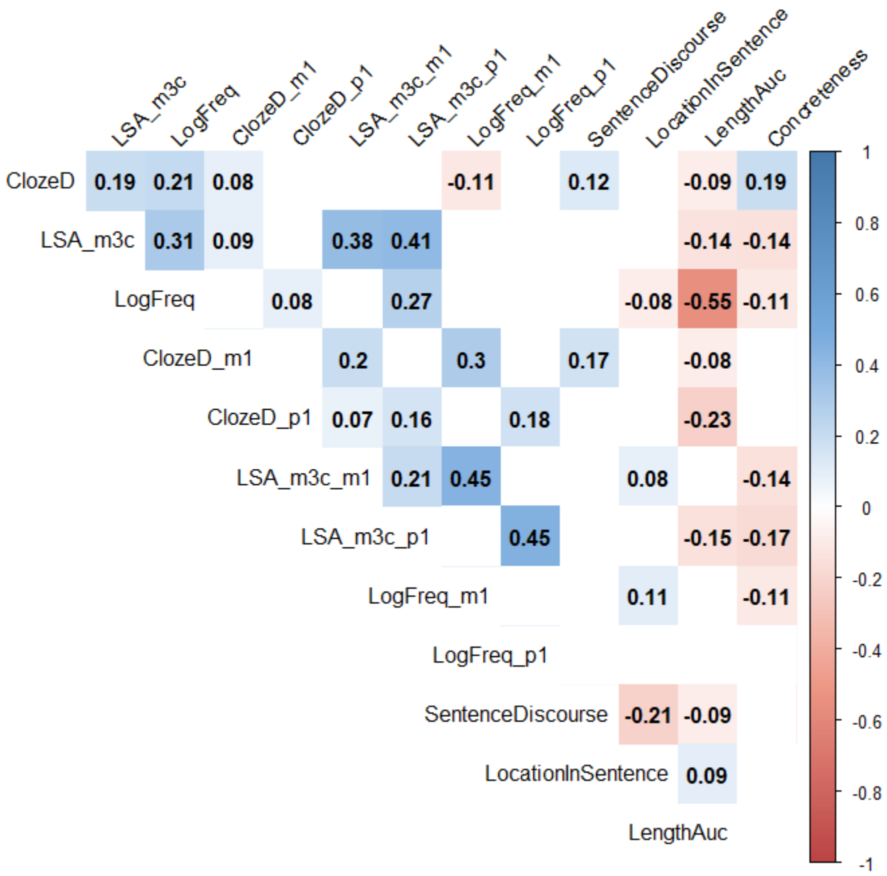

Supplementary Analyses 3: To ensure that our result patterns are not due to the inclusion of proper names specific to the story, processing of number words, or due to the inclusion of words that may be novel to children, we repeated the key analyses presented in the paper using subsets of the data. Below we include results from models containing: 1) main effects of the key predictors and relevant controls, 2) a model that additionally includes all relevant interactions (same as Model 6 in main paper), 3) the best model fit for children, and 4) a model that includes only effects of Discourse Cloze and Log Frequency in adults. Across all analyses, adults and children show robust effects of Discourse Cloze.

a) Excluding Proper Names and Count Words

Supp. Model 1: Model evaluating main effects from all key predictors and control variables, when all proper names and number words are removed from the analyses

**Factor**	Estimate	Std. Error	p-value
(Intercept)	-0.28	0.12	0.02*
Discourse Cloze	0.50	0.13	0.000***
LSA	0.11	0.16	0.50
Log Frequency	0.12	0.14	0.42
Age Group	-0.02	0.11	0.82
Concreteness	0.09	0.12	0.46
Acoustic Length	0.02	0.14	0.86
Location in Sentence	0.02	0.12	0.89
Sentence in Discourse	0.03	0.12	0.78
Log Frequency N-1	-0.09	0.13	0.51
Log Frequency N+1	-0.11	0.13	0.40
LSA N-1	0.19	0.15	0.21
LSA N+1	-0.01	0.17	0.93
Discourse Cloze N-1	-0.07	0.12	0.58
Discourse Cloze N+1	-0.04	0.13	0.73

Supp. Model 2: Model including key predictors and their interactions with age group (equivalent to Model 6 in main paper), when all proper names and number words are removed from the analyses

**Factor**	Estimate	Std. Error	p-value
(Intercept)	-0.28	0.12	0.02*
Discourse Cloze x Age Group	-0.07	0.12	0.57
**Discourse Cloze**	0.50	0.14	0.000***
LSA x Age Group	-0.01	0.13	0.91
LSA	0.11	0.16	0.51
Log Frequency x Age Group	-0.06	0.11	0.60
Log Frequency	0.11	0.14	0.44

Age Group	-0.03	0.11	0.75
Concreteness	0.09	0.12	0.46
Acoustic Length	0.02	0.14	0.86
Location in Sentence	0.01	0.12	0.90
Sentence in Discourse	0.03	0.12	0.78
Log Frequency N-1	-0.09	0.13	0.50
Log Frequency N+1	-0.11	0.13	0.40
LSA N-1	0.19	0.15	0.21
LSA N+1	-0.01	0.17	0.93
Discourse Cloze N-1	-0.07	0.12	0.58
Discourse Cloze N+1	-0.04	0.13	0.73

Supp. Model 3: Model evaluating the effects of Discourse Cloze in children (equivalent to the best fit model for children in main paper), when all proper names and number words are removed from the analyses

**Factor**	Estimate	Std. Error	p-value
(Intercept)	-0.25	0.19	0.19
Discourse Cloze	0.87	0.18	0.000***

Supp. Model 4: Model evaluating the effects of Discourse Cloze and Log Frequency of the prior word in adults (equivalent to the best fit model for adults in main paper), when all proper names and number words are removed from the analyses

**Factor**	Estimate	Std. Error	p-value
(Intercept)	-0.29	0.10	0.004*
Discourse Cloze	0.43	0.11	0.000***
Log Frequency N-1	-0.20	0.09	0.03*

b) Excluding Words not attested before 5 in CHILDES (MacWhinney, 2000; Sanchez et al., 2019)

Supp. Model 9: Model evaluating main effects from all key predictors and control variables, including only those words that are attested in CHILDES before age 5

**Factor**	Estimate	Std. Error	p-value
(Intercept)	-0.36	0.12	0.003**
Discourse Cloze	0.47	0.11	0.000***
LSA	0.04	0.15	0.80
Log Frequency	0.22	0.15	0.13
Age Group	-0.08	0.11	0.46
Concreteness	0.05	0.12	0.68
Acoustic Length	0.03	0.15	0.86
Location in Sentence	-0.03	0.11	0.81

Sentence in Discourse	0.08	0.11	0.46
Log Frequency N-1	-0.07	0.13	0.58
Log Frequency N+1	-0.03	0.13	0.83
LSA N-1	0.14	0.14	0.32
LSA N+1	-0.22	0.15	0.15
Discourse Cloze N-1	-0.05	0.11	0.66
Discourse Cloze N+1	0.04	0.11	0.73

Supp. Model 10: Model including key predictors and their interactions with age group (equivalent to Model 6 in main paper), including only those words that are attested in CHILDES before age 5

**Factor**	Estimate	Std. Error	p-value
(Intercept)	-0.36	0.12	0.003**
Discourse Cloze x Age Group	-0.12	0.10	0.20
**Discourse Cloze**	0.47	0.11	0.000***
LSA x Age Group	0.07	0.11	0.50
LSA	0.04	0.15	0.77
Log Frequency x Age Group	0.03	0.11	0.78
Log Frequency	0.23	0.15	0.12
Age Group	-0.07	0.11	0.49
Concreteness	0.05	0.12	0.68
Acoustic Length	0.02	0.15	0.86
Location in Sentence	-0.03	0.11	0.81
Sentence in Discourse	0.08	0.11	0.46
Log Frequency N-1	-0.07	0.13	0.58
Log Frequency N+1	-0.03	0.13	0.83
LSA N-1	0.14	0.14	0.32
LSA N+1	-0.22	0.15	0.15
Discourse Cloze N-1	-0.05	0.11	0.66
Discourse Cloze N+1	0.04	0.11	0.74

Supp. Model 11: Model evaluating the effects of Log Frequency and Discourse Cloze in children (equivalent to the best fit model for children in main paper), including only those words that are attested in CHILDES before age 5

**Factor**	Estimate	Std. Error	p-value
(Intercept)	-0.29	0.16	0.07
Discourse Cloze	0.80	0.15	0.000***

Supp. Model 12: Model evaluating the effects of Log Frequency and Discourse Cloze in adults, including only those words that are attested in CHILDES before age 5

**Factor**	Estimate	Std. Error	p-value
(Intercept)	-0.37	0.11	0.002**
Discourse Cloze	0.33	0.09	0.000***
Log Frequency N-1	-0.26	0.09	0.004**

c) Excluding Words acquired after 10 (Balota et al., 2007; Kuperman et al., 2012)

Supp. Model 13: Model evaluating main effects from all key predictors and control variables, including only those words that are estimated to be acquired before age 10

**Factor**	Estimate	Std. Error	p-value
(Intercept)	-0.31	0.12	0.01*
Discourse Cloze	0.49	0.11	0.000***
LSA	0.05	0.15	0.74
Log Frequency	0.12	0.15	0.43
Age Group	-0.05	0.12	0.67
Concreteness	-0.04	0.12	0.77
Acoustic Length	-0.04	0.15	0.79
Location in Sentence	0.07	0.11	0.55
Sentence in Discourse	0.11	0.11	0.35
Log Frequency N-1	-0.03	0.13	0.81
Log Frequency N+1	-0.04	0.13	0.73
LSA N-1	0.15	0.14	0.27
LSA N+1	-0.19	0.15	0.21
Discourse Cloze N-1	-0.08	0.11	0.50
Discourse Cloze N+1	-0.06	0.11	0.60

Supp. Model 14: Model including key predictors and their interactions with age group (equivalent to Model 6 in main paper), including only those words that are estimated to be acquired before age 10

**Factor**	Estimate	Std. Error	p-value
(Intercept)	-0.31	0.12	0.02*
Discourse Cloze x Age Group	-0.08	0.10	0.40
**Discourse Cloze**	0.49	0.11	0.000***
LSA x Age Group	0.02	0.12	0.89
LSA	0.05	0.15	0.74
Log Frequency x Age Group	-0.02	0.12	0.84
Log Frequency	0.12	0.15	0.43
Age Group	-0.04	0.12	0.73
Concreteness	-0.04	0.12	0.77
Acoustic Length	-0.04	0.15	0.79
Location in Sentence	0.07	0.11	0.55
Sentence in Discourse	0.11	0.11	0.35
Log Frequency N-1	-0.03	0.13	0.81
Log Frequency N+1	-0.04	0.13	0.73
LSA N-1	0.15	0.14	0.27
LSA N+1	-0.19	0.15	0.21
Discourse Cloze N-1	-0.08	0.11	0.50
Discourse Cloze N+1	-0.06	0.11	0.60

Supp. Model 15: Model evaluating the effects of Log Frequency and Discourse Cloze in children (equivalent to the best fit model for children in main paper), including only those words that are estimated to be acquired before age 10

**Factor**	Estimate	Std. Error	p-value
(Intercept)	-0.28	0.17	0.12
Discourse Cloze	0.77	0.16	0.000***

Supp. Model 16: Model evaluating the effects of Log Frequency and Discourse Cloze in adults, including only those words that are estimated to be acquired before age 10

**Factor**	Estimate	Std. Error	p-value
(Intercept)	-0.30	0.10	0.01**
Discourse Cloze	0.34	0.09	0.000***
Log Frequency N-1	-0.24	0.09	0.01*

Supplementary Analyses 4: To corroborate the null effects of Frequency in children's data in the current paper, we used child-based frequency measures calculated from the CHILDES database (MacWhinney, 2000). Values were calculated based on the number of instances a word was recorded either when spoken by a child or by a parent before the age of 10. We repeated our primary analyses in children with the child-based frequency norms and in none of the models did the effect of Frequency at centro-parietal sites make a significant contribution to model fit ($p > 0.1$).

Supplementary Analyses 5: To corroborate the null effects of LSA in the current paper, we used two additional measures of LSA (Landauer & Dumais, 1997; Landauer et al., 1998). In the first, semantic association was operationalized as the average of the LSA association values of the two words immediately preceding each target word using the General Reading up to 1st Year of College (300 factors) topic space as well as the General Reading up to 3rd Grade (300 factors) topic space. Across all analyses we see no effect of LSA over and above the effects of Frequency (with the exception of an effect at Fz in Children).

Supplementary Analyses 6: The results reveal similar patterns to the intercept models, and minor differences are included in the main paper. Although models converge, maximal models are singular raising worries about over fitting, thus, intercept only models are presented in the paper. In addition, we confirmed our main pattern of results using a more maximal stable model; with random intercepts for Subject and Item as well as random slopes for Cloze Discourse by Subject. Other random effects were removed in order of theoretical importance until all models converged either without a singular fit or, using partially Bayesian modeling. Bayesian computations were conducted using the *blme* package (Dorie, 2015) in R.

Supplementary Analyses 7:

Our confidence in the critical null effects in the current study were tested by computing Bayes Factors. Specifically, for null effects of interest (listed below), we evaluated the Bayes Factor in favor of the more maximal model, which included the factor of interest (H_1) relative to the null model, with the factor of interest removed (H_0). Bayes Factors were computed using the *brms* package in R (Bürkner, 2017) and used values of 2,000 for warm-up and 20,000 iteration phases of the analysis. A Bayes Factor of 1-3 was considered weak evidence in favor of H_1 and values >3 were considered strong evidence in favor of H_1. Bayes Factor less than 1 were considered evidence in favor of the null hypothesis, H_0. <u>Thus, a BF less than 1 indicated support for the critical null effect.</u>

In order to evaluate Bayes Factors with consideration for plausible prior distributions we conducted a sensitivity analysis. The prior for each effect of interest was set as a normal distribution with a mean of 0. We then evaluated Bayes Factors for models (H_1s) with a range of standard deviations designed to represent plausible effect sizes.

We estimated standard deviations of our priors based on our strongest detected ERP effect size; the effect of Discourse Cloze (from Model 6). The effect size of Discourse Cloze was ~0.47. Thus, we considered the following standard deviations; 0.5, 0.15, 0.25, 0.35, 0.45. For all other parameters within the model, we used *brms* default priors.

Results:

Null Effect of Cloze x Age Group Interaction

As can be seen in Figure 1, Bayes Factors analysis showed evidence in favor of a null interaction between Cloze Discourse x Age Group for the N400 (Model 6). Although the amount of evidence only weakly supported the null (>0.3), evidence was in favor (<1) of the Null Hypothesis for all effect sizes with the exception of the smallest (0.05), which favored neither H_0 nor H_1.

Figure 1:

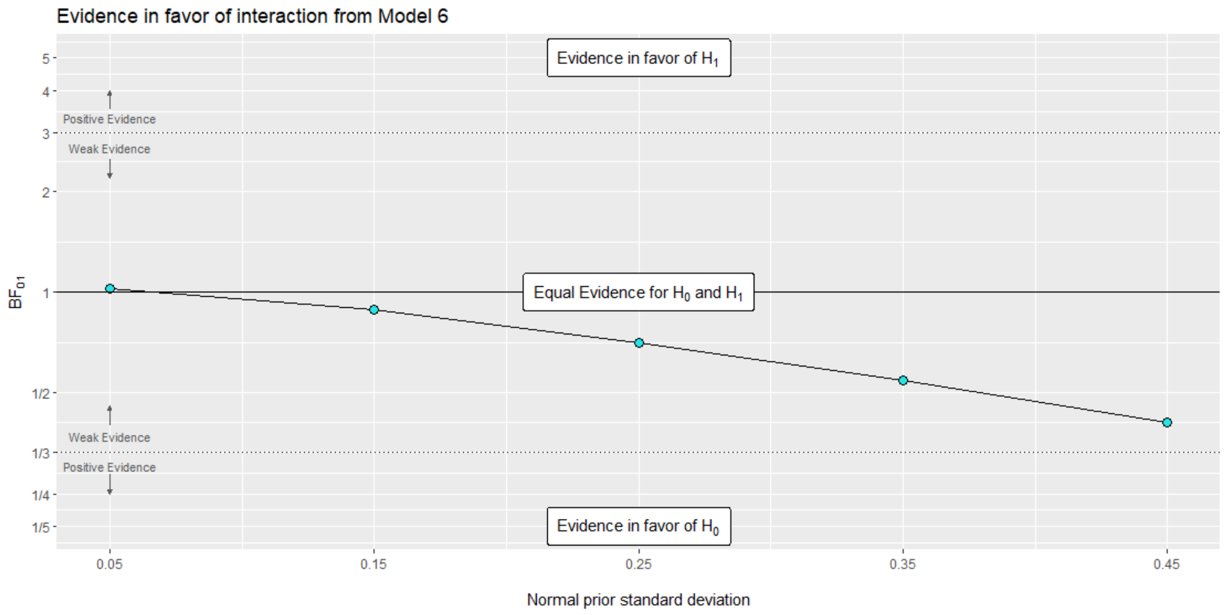

Null Effect of Frequency in Children

Bayes Analyses showed mixed evidence regarding the null effect of Frequency on the N400 response in Children. As can be seen in Figure 2, Bayes Factors wavered right around 1 across effect sizes. This is unsurprising, as the effect of Frequency in Children is significant at anterior electrode sites and may extend into more anterior sites of the canonical N400 response. In addition, although the effect of Frequency did not reach significance in Children's N400 response (p = 0.14) the effect of Frequency was marginal in the combined Adult and Child analyses (Model 1).

Figure 2:

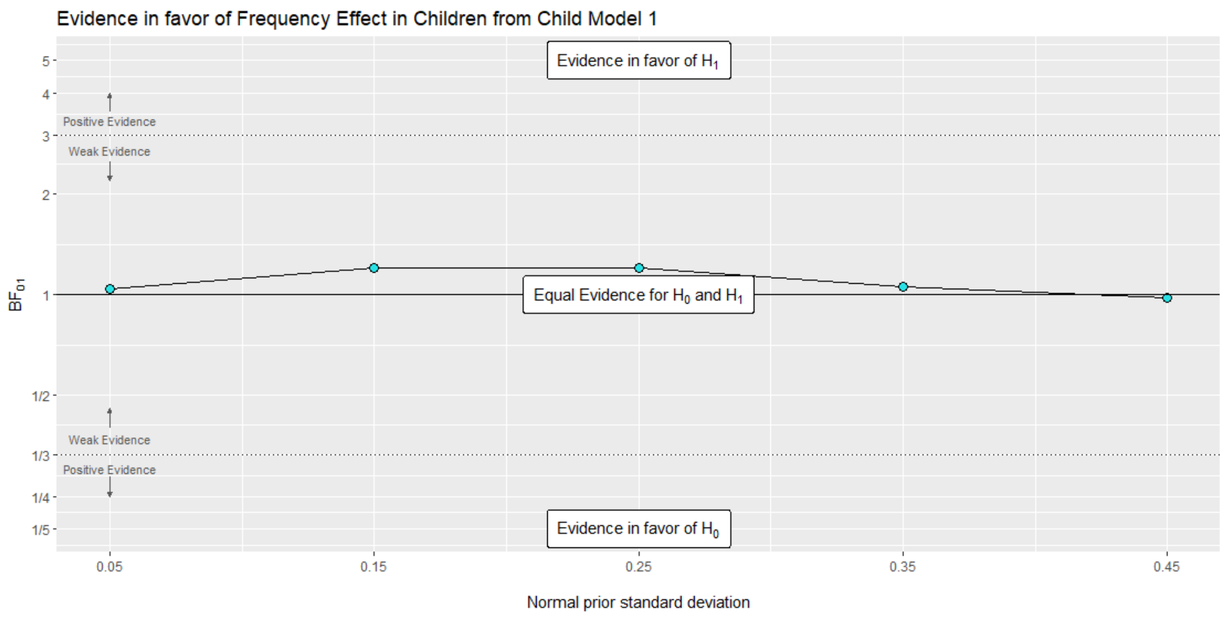

In addition, have calculated Bayes Factors for all other null analyses using the BayesFactor package in R (Morey et al., 2015). All Bayes Factor analyses were conducted using default priors specified on a normalized scale. Importantly, all but one of the null effects showed Bayes Factors < 0.33, suggesting substantial evidence in favor of the null hypothesis. The only exception was the Bayes Factor for the null effect of Frequency in Adult participants, which had a Bayes Factor of 0.40, suggesting only anecdotal evidence for the null hypothesis. This is unsurprising considering the significant anterior effects of frequency which may have influenced more anterior locations within the N400 response.

References:

Balota, D. A., Yap, M. J., Hutchison, K. A., Cortese, M. J., Kessler, B., Loftis, B., Neely, J. H., Nelson, D. L., Simpson, G. B., & Treiman, R. (2007). The English lexicon project. *Behavior Research Methods*, *39*, 445-459.

Bürkner, P.-C. (2017). brms: An R package for Bayesian multilevel models using Stan. Journal of statistical software, 80, 1-28.

Dorie, V. (2015). blme: Bayesian Linear Mixed-Effects Models. R package version 1.0-4. In.

Kuperman, V., Stadthagen-Gonzalez, H., & Brysbaert, M. (2012). Age-of-acquisition ratings for 30,000 English words. *Behavior Research Methods*, *44*, 978-990.

Landauer, T. K., & Dumais, S. T. (1997). A solution to Plato's problem: The latent semantic analysis theory of acquisition, induction, and representation of knowledge. *Psychological review*, *104*(2), 211.

Landauer, T. K., Foltz, P. W., & Laham, D. (1998). An introduction to latent semantic analysis. *Discourse processes*, *25*(2-3), 259-284.

MacWhinney, B. (2000). *The CHILDES Project: Tools for analyzing talk. transcription format and programs* (Vol. 1). Psychology Press.

Morey, R. D., Rouder, J. N., Jamil, T., & Morey, M. R. D. (2015). Package ‘bayesfactor’. URL http://cran/r-projectorg/web/packages/BayesFactor/BayesFactor pdf i (accessed 1006 15).

Sanchez, A., Meylan, S. C., Braginsky, M., MacDonald, K. E., Yurovsky, D., & Frank, M. C. (2019). childes-db: A flexible and reproducible interface to the child language data exchange system. *Behavior Research Methods*, *51*(4), 1928-1941. https://link.springer.com/content/pdf/10.3758/s13428-018-1176-7.pdf
